# Genome-wide changes in microRNA expression during short and prolonged heat stress and recovery in contrasting rice cultivars

**DOI:** 10.1093/jxb/erx111

**Published:** 2017-04-12

**Authors:** Satendra K. Mangrauthia, Sailaja Bhogireddy, Surekha Agarwal, Vishnu V. Prasanth, S. R. Voleti, Sarla Neelamraju, Desiraju Subrahmanyam

**Affiliations:** 1 ICAR-Indian Institute of Rice Research, Hyderabad 500030,India

**Keywords:** High temperature, miRNA, NGS, *Oryza sativa*, oxidative stress, transcriptome.

## Abstract

MicroRNAs (miRNAs) are known to regulate expression of genes under stress. We report here the deep sequencing of small RNAs expressed during control, short and prolonged heat stress and recovery. Genome-wide identification of miRNAs in tolerant (Nagina 22) and susceptible (Vandana) rice cultivars was performed in 16 samples representing root and shoot of 13-day-old seedlings. The expression profile of miRNAs was analysed in 36 pairwise combinations to identify the genotype-, treatment- and tissue-dependent expression of miRNAs. Small-RNA sequencing of 16 libraries yielded ~271 million high-quality raw sequences; 162 miRNA families were identified. The highly expressed miRNAs in rice tissues were miR166, miR168, miR1425, miR529, mR162, miR1876, and miR1862. Expression of osa-miR1436, osa-miR5076, osa-miR5161, and osa-miR6253 was observed only in stressed tissue of both genotypes indicating their general role in heat stress response. Expression of osa-miR1439, osa-miR1848, osa-miR2096, osa-miR2106, osa-miR2875, osa-miR3981, osa-miR5079, osa-miR5151, osa-miR5484, osa-miR5792, and osa-miR5812 was observed only in Nagina 22 during high temperature, suggesting a specific role of these miRNAs in heat stress tolerance. This study provides details of the repertoire of miRNAs expressed in root and shoot of heat susceptible and tolerant rice genotypes under heat stress and recovery.

## Highlight

Heat susceptible and tolerant rice genotypes have different landscapes of miRNAs and the tolerant genotypes show efficient recovery mediated by miRNAs. Roots regulate miRNAs more sensitively than shoots during heat stress.

## Introduction

Plants exposed to stress use multiple gene regulatory mechanisms, including post-transcriptional regulation of gene expression, to restore and re-establish cellular homeostasis. MicroRNAs (miRNAs) have emerged as ubiquitous critical regulator molecules in nearly all eukaryotes (Bartel, 2004). They are a group of small endogenous non-coding RNAs (19–25 nucleotides) that negatively modulate gene expression at the post-transcriptional levels by directing the cleavage of target genes or by inhibiting translation depending on the extent of the complementarity between the miRNA and its target (Reinhart *et al.*, 2002; Chen, 2004; Jones-Rhoades and Bartel, 2004).

Plant miRNAs have diverse functions in growth and development processes, including root development (Guo *et al.*, 2005), leaf morphogenesis (Kim *et al.*, 2005), transition of growth from vegetative to reproductive stage (Achard *et al.*, 2004; Lauter *et al.*, 2005), and floral development and differentiation (Chen, 2004). The association of miRNAs with abiotic stress responses is now well established (Sunkar and Zhu, 2004; Jeong *et al.*, 2011; May *et al.*, 2013; Agarwal *et al.*, 2015), but despite miRNAs being the key components of gene regulation during these stresses, the study of high temperature-responsive miRNAs is very limited in rice and other crops (Jeong and Green, 2013). Understanding miRNA expression and regulation during elevated temperature will help in better understanding the molecular pathways associated with high temperature response in the model monocot crop rice. The use of miRNAs for trait improvement has been successfully demonstrated wherein overexpression of OsmiR397 enhanced rice yield by increasing grain size and promoting panicle branching (Zhang *et al.*, 2013).

 In this study, 16 small RNA libraries were prepared from root and shoot tissue of heat susceptible and tolerant rice genotypes. Two contrasting rice genotypes, Nagina 22 (N22) as tolerant and Vandana as susceptible, were selected based on earlier reports (Jagadish *et al.*, 2008; Sailaja *et al.*, 2014, 2015). The libraries represented small RNAs expressed during optimum temperature (control), high temperature (short and prolonged stress) and recovery. These libraries were sequenced to identify the miRNAs expressed in shoot and root of rice genotypes in control and stressed environments. The target genes of these miRNAs were predicted. Expression of miRNAs and target genes was verified using quantitative PCR.

## Materials and methods

### Plant materials and heat stress treatments

Rice (*Oryza sativa*) seeds of cultivars N22 and Vandana were used for small RNA sequencing and miRNA analysis. The details of germination, growth conditions and heat stress treatments are the same as described previously (Sailaja *et al.*, 2014). Germinated seedlings were transferred to Yoshida medium (Yoshida *et al.*, 1976) and maintained at 13 h of light and 11 h dark at optimum day/night temperature (30 °C/24 °C). Eight-day-old seedlings were subjected to high temperature (42 °C/36 °C day/night) treatments for 24 h (short) and 5 days (long). To get the recovery samples, high temperature-treated plants (for 4 days) were shifted back to optimum temperature for 24 h. In total, 16 samples were collected from root and shoot of N22 and Vandana representing control (30 °C/24 °C), short duration heat stress (SDS) treatment (42 °C/36 °C day/night for 24 h), long duration heat stress (LDS) treatment (42 °C/36 °C day/night for 5 days), and 24 h of recovery (REC) at optimum temperature (30 °C/24 °C) after a heat stress treatment (42 °C/36 °C) of 4 days. On the 13th day after germination, the 16 samples were harvested from four batches (control, SDS, LDS, and REC) including root and shoot tissues of N22 and Vandana.

### Sequencing of small RNA

Total RNAs were extracted from 16 samples using TRIzol Reagent (Life Technologies). RNA was isolated separately from root and shoot tissues subjected to control and stress treatments. Small RNA enriched samples were sequenced using Illumina sequencing. Samples of both the cultivars were harvested from two biological replicates. The small RNA quality statistics were generated using FASTX Toolkit (v 0.0.13) (http://hannonlab.cshl.edu/fastx_toolkit/). The quality box plot graph was generated to visualize the quality score of each base with respect to its base position. Quality filtering was performed using the fastq quality filter program, and quality cutoff (q) of 20 and minimum percentage (p) value of 100 were assigned for preprocessing. Raw data were subjected to quality filtering (q20/p100) and the resultant reads were retained. During the quality filtering process, the low quality reads were discarded from the total reads. Adapter clipping was performed for those high quality (HQ) score reads using the FASTX toolkit. The sequence length distribution of HQ score reads was analysed individually using the FastQC tool (http://www.bioinformatics.babraham.ac.uk/projects/fastqc/). The sequence data have been submitted to NCBI. The BioProject ID is PRJNA322758.

### Analyses of miRNAs

**Fig. 1.**
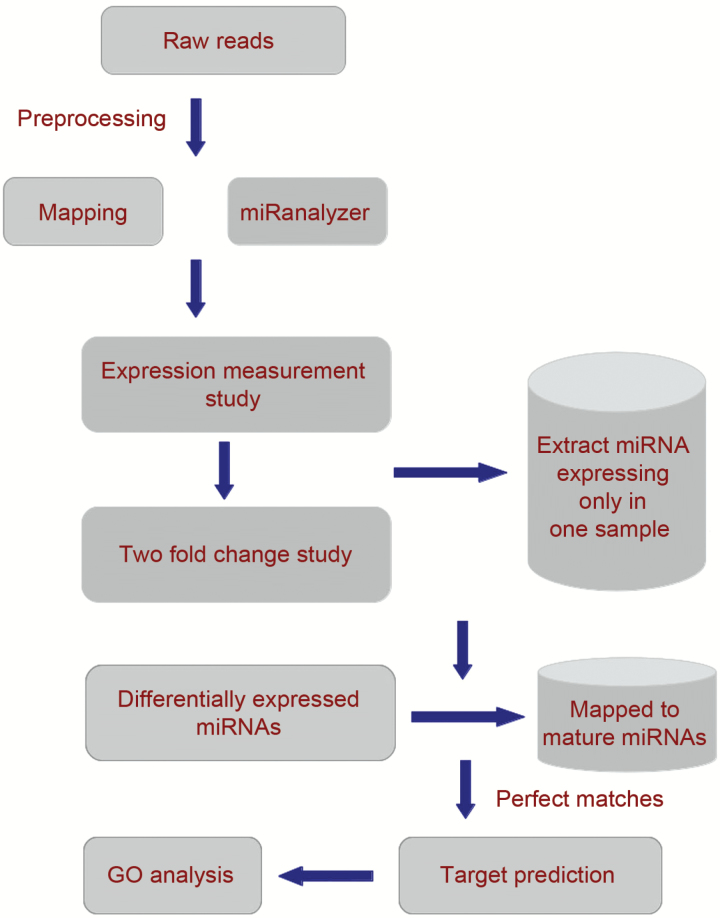
The outline of the bioinformatics analysis work flow followed for identification of miRNAs in rice. (This figure is available in color at *JXB* online.)

The outline of the bioinformatics analysis work flow is given in Fig. 1. The *O. sativa* Nipponbare reference genome was downloaded from the MSU Rice Genome Annotation Project Database and Resource (Kawahara *et al.*, 2013). The HQ score reads of 16 samples of *O. sativa* were mapped to the reference genome of *O. sativa* Nipponbare using bowtie (Langmead *et al.*, 2009). The HQ score reads were mapped to miRNAs of *O. sativa* using bowtie (Langmead *et al.*, 2009). To detect the miRNAs, the miRanalyzer standalone version (Hackenberg *et al.*, 2011) was used. The number of mismatches allowed for alignment to known miRNAs, transcribed libraries and genome was set to zero. For several pairwise combinations, the differentially transcribed miRNAs with expression exhibiting two-fold or higher were chosen for downstream analysis. Gitools was used for drawing heat maps of differentially expressed miRNAs (Perez-Llamas and Lopez-Bigas, 2011).

### Target prediction of miRNAs and function analysis

The target prediction of miRNAs was performed using the plant miRNA target prediction program psRNATarget (Dai and Zhao, 2011). To study Gene Ontology (GO), the best target genes were picked. Also the GO Slim term from the MSU Rice Genome Annotation Project Database and Resource (Kawahara *et al.*, 2013) was downloaded for downstream analysis. The target genes were mapped to the GO Slim term using in-house perl script.

### Quantitative real-time PCR

For quantification of miRNAs, small RNA was isolated using mirVana™ miRNA Isolation Kit (cat. no. AM1560, Ambion). The reverse transcription (RT) of miRNAs was performed using miScript II RT Kit (cat. no. 218161, Qiagen). The RT reaction was prepared by mixing and incubating the template small RNAs, miScript Reverse Transcriptase Mix, 10× miScript Nucleics Mix, and 5× miScript HiSpec Buffer, as specified by the manufacturer. Using miScript II RT Kit, mature miRNAs were polyadenylated by poly(A) polymerase and reverse transcribed into cDNA using oligo-dT primers. The oligo-dT primers have a 3′ degenerate anchor and a universal tag sequence on the 5′ end that allows amplification of mature miRNA during the real-time PCR step. cDNA was normalized and used for real time PCR by miScript SYBR Green PCR Kit (cat. no. 218073, Qiagen), which contains the miScript Universal Primer (reverse primer) and QuantiTect SYBR Green PCR Master Mix. The real time PCR reaction was prepared by mixing 2× QuantiTect SYBR Green PCR Master Mix, 10× miScript Universal Reverse Primer, miRNA specific forward primer (Supplementary Table S1 at *JXB* online), and template cDNA, as specified by the kit manufacturer. U6 was used as internal control for miRNA quantification (Ding *et al.*, 2011). The quantitative real-time PCR (qRT-PCR) temperature profile was followed as described previously (Sailaja *et al.*, 2014).

For quantification of target genes, mRNA was isolated from the same plant samples using mirVana™ miRNA Isolation Kit as the kit provides an option for isolating small RNAs and long RNAs from the same preparation by adjusting the ethanol concentration. cDNA synthesis of mRNAs was performed using the Improm-II reverse transcription system, as specified by the manufacturer (Promega, cat. no. A3800). For cDNA synthesis, oligo dT primers were used. cDNA was treated with RNase and normalized for equal concentration. qRT-PCR was performed using SYBR Premix Ex-Taq (Takara, cat. no. RR820A) in an ABI GeneAmp 7500 Sequence Detection System. *OsActin1* (Lee *et al.*, 2011) was used as the internal control for quantification of genes. Forward and reverse primers used for qRT-PCR of target genes are listed in Supplementary Table S1. Amplification conditions were followed as described previously (Sailaja *et al.*, 2014).

With three biological replications, each reaction was run in duplicate and the melting curves were constructed using Dissociation Curves Software (Applied Biosystems) to ensure that only a single specific product was amplified. The comparative threshold cycle (*C*_T_) method was used to quantify the relative expression levels of miRNAs and genes in real-time PCR. Δ*C*_T_ was calculated by *C*_T_ target-*C*_T_ reference (internal control). Further ΔΔ*C*_T_ values were calculated by Δ*C*_T_ of the stress sample − Δ*C*_T_ of the control sample, and then the fold difference was calculated from 2^−ΔΔ*C*T^. Similarly, the Δ*C*_T_ standard deviation was calculated as given at http://www.appliedbiosystems.com/absite/us/en/home/support/tutorials/realtime-pcr-trouble-shooting-guide.html.

## Results

**Fig. 2.**
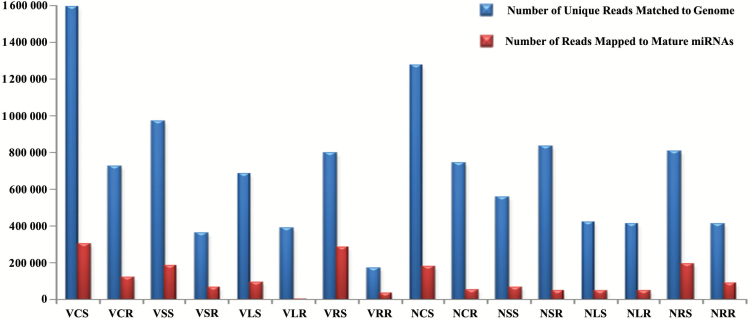
Number of small RNA sequence reads matched to miRNA sequences and unique reads matched to rice genome. Number of reads of 16 small RNA libraries obtained from root and shoot of N22 and Vandana are shown. (This figure is available in color at *JXB* online.)

We used the massively parallel high throughput sequencer to investigate the genome-wide identification and expression profiles of miRNAs in Vandana and N22 rice cultivars, particularly for the heat stress-responsive miRNAs. Sixteen small RNA libraries were constructed by the use of total RNAs isolated from control shoot (CS), control root (CR), short duration stress (SDS) shoot (SS), SDS root (SR), long duration stress (LDS) shoot (LS), LDS root (LR), recovery shoot (RS), and recovery root (RR) tissues of both the cultivars. Small-RNA sequencing of 16 libraries yielded a total of 319 260 462 raw reads including of 271 353 814 high-quality raw sequences (Table 1). The high-quality (HQ) sequence reads of 16 samples of *O. sativa* extracted from the shoot and root in different conditions (control, stress, prolong stress and recovery) were mapped to the reference genome of *O. sativa* Nipponbare using bowtie (Langmead *et al.*, 2009) (Table 1). Further, the HQ score reads were mapped to miRNAs of *O. sativa* using bowtie (Langmead *et al.*, 2009). As shown in Fig. 2, the highest abundance of miRNAs was found in the shoot (control) library of Vandana. In the N22 libraries, RS showed the maximum number of miRNA reads. The length distribution of sRNA reads revealed that heat stress treatments affect small RNA metabolism extensively. The sRNA class of 24 nt was the most abundant group in the control sample of each library followed by recovery samples (Supplementary Fig. S1). Stressed tissues showed variation in terms of sequence length.

**Table 1.** Summary of 16 small RNA libraries prepared from control and stressed root and shoot tissue of rice genotypes N22 and Vandana

**Sample**	**Total number of reads**	**Total number of reads after quality filtering**	**Total number of reads after adapter removal**	**Percentage mapped to rice genome**	**Number of reads mapped to mature miRNAs**	**Number of mature miRNAs detected**
Vandana-CS	22 317 379	19 490 983	19 485 806	94.44	3,05 917	170
Vandana-CR	18 747 317	16 485 727	16 477 372	29.32	122 238	122
Vandana-LS	21 031 229	18 811 506	18 802 901	89.45	93 220	140
Vandana-LR	15 081 038	13 617 794	12 672 421	23.65	3582	39
Vandana-SS	17 220 411	14 505 821	14 503 031	93.12	183 772	166
Vandana-SR	17 893 454	14 854 505	14 728 017	36.02	68 992	109
Vandana-RS	17 301 437	14 652 170	14 643 846	84.84	285 673	171
Vandana-RR	14 950 788	13 187 788	12 672 491	33.51	37 037	82
N22-CS	18 585 785	15 288 386	15 284 870	90.28	179 590	173
N22-CR	16 191 258	14 265 982	14 251 351	54.71	53 353	126
N22-LS	20 724 912	18 400 120	18 397 168	97.52	47 762	120
N22-LR	21 260 419	18 251 696	18 247 384	41.66	48 680	114
N22-SS	13 173 663	10 663 589	9 418 764	90.9	69 358	129
N22-SR	40 196 517	32 504 289	30 436 715	78.54	50 469	154
N22-RS	25 503 799	20 808 814	20 804 542	93.76	196 664	152
N22-RR	19 081 056	15 564 644	15 557 190	27.65	91 613	108

### Identification of miRNAs in *Oryza sativa*

In order to identify conserved and species-specific miRNAs from control and high temperature-exposed root and shoot tissues of N22 and Vandana, small RNA sequences generated from each library were independently aligned with known and experimentally validated mature miRNAs of *O. sativa* deposited in miRBase. After homology search, a minimum of 39 miRNAs (Vandana-LR) and a maximum of 173 miRNAs (N22-CS) were detected in 16 small RNA libraries (Table 1). These miRNAs belong to 162 miRNA families (Supplementary Table S2), suggesting wide occurrence of miRNA-mediated gene regulation under heat stress. Out of 162 families of miRNA, 33 showed shoot preferential expression, 12 showed root preferential expression, and 117 showed expression in both the tissues. In order to identify cultivar preferential miRNAs, N22 and Vandana miRNAs were compared. N22 showed expression of 144 miRNA families while Vandana showed expression of 141. Of these, 21 families showed N22 preferential expression, 18 families showed Vandana preferential expression (Table 2), and 123 families showed expression in both the cultivars. Further, miRNA families showing expression preferentially in control and stressed tissues were identified in both the genotypes (Table 3) and their target genes were predicted (Supplementary Table S3). Expression of osa-miR1319 and osa-miR5809 was recorded only in control tissue while expression of osa-miR1436, osa-miR5076, osa-miR5161, and osa-miR6253 was observed only in heat-treated tissue (SDS and LDS) of both genotypes.

**Table 2.** List of miRNA families showing tissue and cultivar preferential expression based on this study

**Tissue or cultivar**	**MicroRNA families**
Shoot preferential miRNAs	osa-miR1317/1882e, osa-miR1429, osa-miR1439, osa-miR172, osa-miR1852, osa-miR1856, osa-miR1864, osa-miR1875, osa-miR1880, osa-miR2096, osa-miR2864, osa-miR2875, osa-miR2905, osa-miR395, osa-miR5073, osa-miR5076, osa-miR5079, osa-miR5158, osa-miR5162, osa-miR530, osa-miR5339, osa-miR5501, osa-miR5502, osa-miR5503, osa-miR5510, osa-miR5525, osa-miR5537, osa-miR5542, osa-miR5792, osa-miR5809, osa-miR5811, osa-miR5825, osa-miR5155
Root preferential miRNAs	osa-miR1854, osa-miR1870, osa-miR1872, osa-miR1873, osa-miR2055, osa-miR2866, osa-miR2872, osa-miR2876, osa-miR3981, osa-miR5484, osa-miR5153
N22 preferential miRNAs	osa-miR1427, osa-miR1439, osa-miR1848, osa-miR1852, osa-miR1865, osa-miR1872, osa-miR2096, osa-miR2106, osa-miR2875, osa-miR395, osa-miR3981, osa-miR5153, osa-miR5155, osa-miR530, osa-miR5484, osa-miR5502, osa-miR5537, osa-miR5542, osa-miR5792, osa-miR5823, osa-miR5815
Vandana preferential miRNAs	osa-miR1317, osa-miR1429, osa-miR1431, osa-miR1854, osa-miR1856, osa-miR1864, osa-miR2864, osa-miR2874, osa-miR2876, osa-miR2880, osa-miR414, osa-miR531, osa-miR5501, osa-miR5503, osa-miR5510, osa-miR5525, osa-miR5803, osa-miR5811

**Table 3.** MicroRNA families showing control and stress preferential expression in N22 and Vandana

**miRNA families expressed preferentially in control tissue**	**miRNA families expressed preferentially in stressed tissue**
osa-miR1317, osa-miR1319, osa-miR172, osa-miR1852, osa-miR1856, osa-miR1864, osa-miR2866, osa-miR395, osa-miR399, osa-miR5073, osa-miR5155, osa-miR5158, osa-miR5339, osa-miR5502, osa-miR5523, osa-miR5537, osa-miR5538, osa-miR5799, osa-miR5801, osa-miR5804, osa-miR5809, osa-miR5809, osa-miR5811, osa-miR5823, osa-miR6248	osa-miR1318, osa-miR1429, osa-miR1436, osa-miR1439, osa-miR1848, osa-miR1854, osa-miR1869, osa-miR1873, osa-miR2096, osa-miR2106, osa- miR2870, osa-miR2875, osa-miR2877, osa-miR319, osa-miR3981, osa-miR5076, osa-miR5077, osa-miR5079, osa-miR5151, osa-miR5158, osa-miR5160, osa-miR5161, osa-miR5484, osa-miR5493, osa-miR5501, osa-miR5503, osa- miR5792, osa-miR5812, osa-miR6253, osa-miR6253
**miRNA families expressed preferentially in control tissue of Vandana**	**miRNA families expressed preferentially in stressed tissue of Vandana**
osa-miR1317, osa-miR1319, osa-miR1856, osa-miR1864, osa-miR399, osa-miR5073, osa-miR5523, osa-miR5538, osa-miR5799, osa-miR5801, osa- miR5804, osa-miR5809, osa-miR5811, osa-miR6248	osa-miR6253, osa-miR5076, osa-miR5077, osa-miR5158, osa-miR5160, osa-miR5161, osa-miR5493, osa-miR5501, osa-miR5503, osa-miR1869, osa- miR1873, osa-miR2870, osa-miR2877, osa-miR319, osa-miR1429, osa-miR1436, osa-miR1854
**miRNA families expressed preferentially in control tissue of N22**	**miRNA families expressed preferentially in stressed tissue of N22**
osa-miR1319, osa-miR172, osa-miR1852, osa-miR2866, osa-miR395 osa-miR5155, osa-miR5158, osa-miR5339, osa-miR5502, osa-miR5537, osa-miR5809, osa-miR5823	osa-miR1318, osa-miR1436, osa-miR1439, osa-miR1848, osa-miR2096, osa-miR2106, osa-miR2875, osa-miR3981, osa-miR5076, osa-miR5079, osa- miR5151, osa-miR5161, osa-miR5484, osa-miR5792, osa-miR5812, osa-miR6253
**miRNA families expressed preferentially in control tissue of both the genotypes**	**miRNA families expressed preferentially in stressed tissue of both the genotypes**
osa-miR1319 (Vandana root and N22 shoot), osa-miR5809 (N22 and Vandana shoot)	osa-miR1436 (Vandana shoot and N22 root-SDS), osa-miR5076 (Vandana and N22 shoot-SDS), osa-miR5161 (Vandana shoot and N22 root-SDS), osa-miR6253 (N22 shoot and root-SDS and LDS, Vandana root-SDS)

The expression levels varied from miRNA to miRNA and from library to library ranging from one copy to more than 200 000 copies (Supplementary Table S4). As reported previously, evolutionarily conserved miRNAs have generally high expression abundance when compared with non-conserved miRNAs. The highly expressing miRNAs included miR166, miR168, miR1425, miR529, mR162, miR1876, and miR1862. Of these, miR166 was the most abundant miRNA in control and heat-treated libraries of both N22 and Vandana. Further, N22 showed abundance of miR2877 in all the libraries. Root tissue of N22 showed more abundance of miR396 than shoot.

### Genome-wide expression analysis of heat-responsive miRNAs in *Oryza sativa*

MicroRNAs identified in each library were compared in 36 pairwise combinations to decipher differentially expressed miRNAs. The expression value of each rice miRNA was analysed and miRNAs showing >2.0-fold expression are listed (Supplementary Table S5). The target prediction of differentially expressed miRNAs was performed and the list of target genes is provided (Supplementary Table S6).

**Fig. 3.**
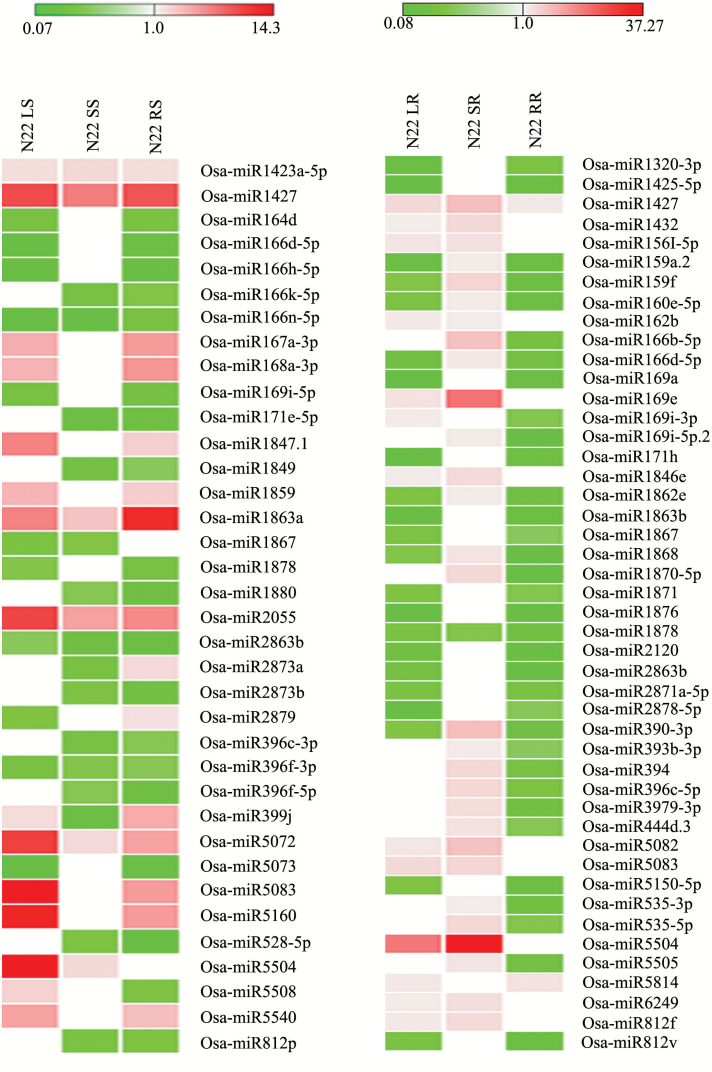
Heat map showing expression of miRNAs in shoot (left side) and root (right side) tissue of N22 after heat stress treatments and recovery. The expression of miRNAs was compared with control sample of respective tissue. The gaps indicate no expression. The values shown in bars at the top indicate fold change regulation in heat-treated samples in comparison with control. (This figure is available in color at *JXB* online.)

Heat-treated shoot tissue of N22 was compared with control shoot. Here, osa-miR1423a-5p, osa-miR1427, osa-miR2055, osa-miR1863a, and osa-miR5072 showed up-regulation, while osa-miR166n-5p, osa-miR2863b, and osa-miR396f-3p were down-regulated after SDS, LDS, and REC. The common and differentially expressed miRNAs of N22 shoot after heat stress treatments and recovery are shown in Fig. 3. In a similar way, heat-treated root tissue of N22 was compared with control root. Here, osa-miR1427 was up-regulated while osa-miR1878 was down-regulated after SDS, LDS, and REC. Interestingly, the majority of the miRNAs showed down-regulation in root of N22 after LDS and REC. MicroRNAs of N22 root showing common and differential expression after SDS, LDS, and REC are shown in Fig. 3.

**Fig. 4.**
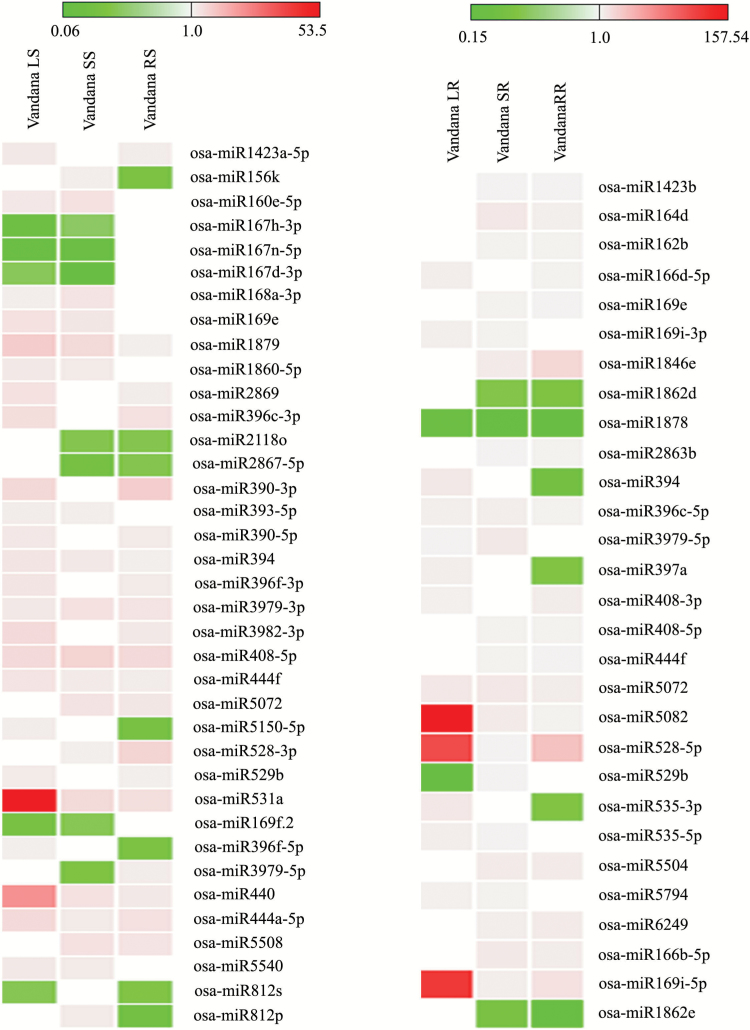
Heat map showing expression of miRNAs in shoot (left side) and root (right side) tissue of Vandana after heat stress treatments and recovery. The expression of miRNAs was compared with control sample of respective tissue. The gaps indicate no expression. The values shown in bars at the top indicate fold change regulation in heat-treated samples in comparison with control. (This figure is available in color at *JXB* online.)

High temperature-treated shoot tissue of the susceptible cultivar Vandana was also compared with control shoot. Here, osa-miR1879, osa-miR394, osa-miR3979-3p, osa-miR408-5p, osa-miR444f, osa-miR531a, osa-miR440, and osa-miR444a-5p showed up-regulation after SDS, LDS, and REC. The common and differentially expressed miRNAs of Vandana shoot after heat stress treatments and recovery are shown in Fig. 4. Likewise, heat-treated root tissue of Vandana was compared with control root. Here, osa-miR396c-5p, osa-miR5072, osa-miR5082, osa-miR528-5p, and osa-miR169i-5p were up-regulated, while osa-miR1878 showed down-regulation after SDS, LDS, and REC. MicroRNAs of Vandana root showing common and differential expression after SDS, LDS, and REC are shown in Fig. 4. The majority of miRNAs showed up-regulation in root and shoot of Vandana after SDS, LDS, and REC. In addition to differentially expressed miRNAs, there were many miRNAs that showed expression preferentially in one tissue or treatment and were absent in the other; these were also identified (Supplementary Table S7).

### Expression analysis of microRNAs in N22 *versus* Vandana

**Fig. 5.**
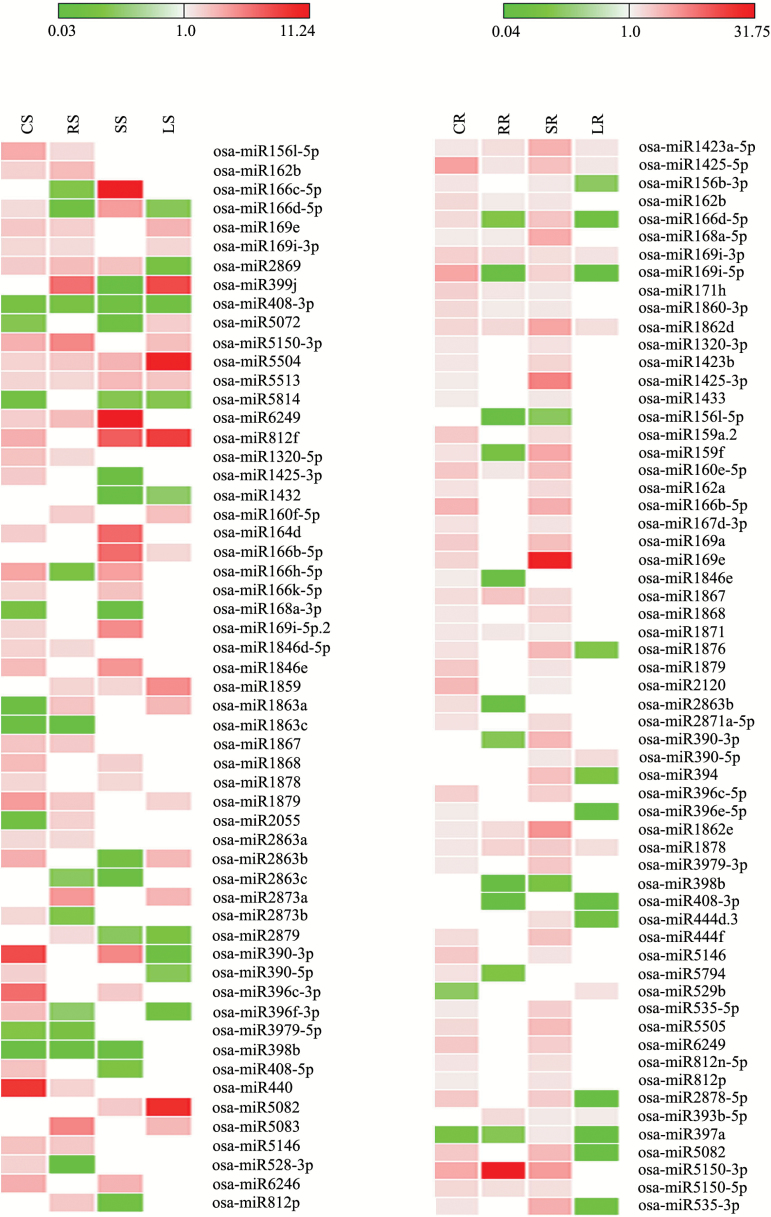
Heat map showing expression of miRNAs in heat tolerant N22 in comparison with heat susceptible Vandana (left side, shoot; right side, root). The comparison was made between two respective samples of N22 and Vandana. The gaps indicate no expression. The values shown in bars at the top indicate fold change regulation in N22 in comparison with Vandana. (This figure is available in color at *JXB* online.)

In addition to comparing the miRNA expression of heat-treated tissue with control tissue of the respective genotype, we compared the expression of miRNAs between tolerant and susceptible rice genotypes. The miRNA expression in heat tolerant N22 was compared with the respective tissue of heat susceptible Vandana. The expression pattern of miRNAs in N22 (in comparison with Vandana) is shown in Fig. 5. In comparison with Vandana shoot, N22 shoot showed higher expression of osa-miR5504 and osa-miR5513, and decreased expression of osa-miR408-3p in all the four samples (control, SDS, LDS, and REC). osa-miR1859 showed higher expression in SDS, LDS, and REC. Similar to shoot, miRNA expression in root tissue of both the genotypes was compared. Root tissue of N22 showed higher expression of osa-miR1423a-5p, osa-miR1425-5p, osa-miR169i-3p, osa-miR1862d, and osa-miR1878 in control, SDS, LDS, and REC. osa-miR160e-5p, osa-miR162b, osa-miR168a-5p, osa-miR171h, osa-miR1860-3p, osa-miR1862e, osa-miR1867, osa-miR1871, osa-miR5150-3p, and osa-miR5150-5p showed higher expression in control, SDS, and REC of N22 root. osa-miR393b-5p showed higher expression in SDS, LDS, and REC of N22 root. A large number of miRNAs showed higher expression in N22-SR than Vandana-SR suggesting that root was more responsive in gene regulation during heat stress. LDS and REC root of N22 showed decreased expression of osa-miR166d-5p, osa-miR169i-5p, osa-miR397a, and osa-miR408-3p, while SDS and REC root of N22 showed decreased expression of osa-miR156l-5p and osa-miR398b.

### Expression validation of miRNAs and target genes

**Fig. 6.**
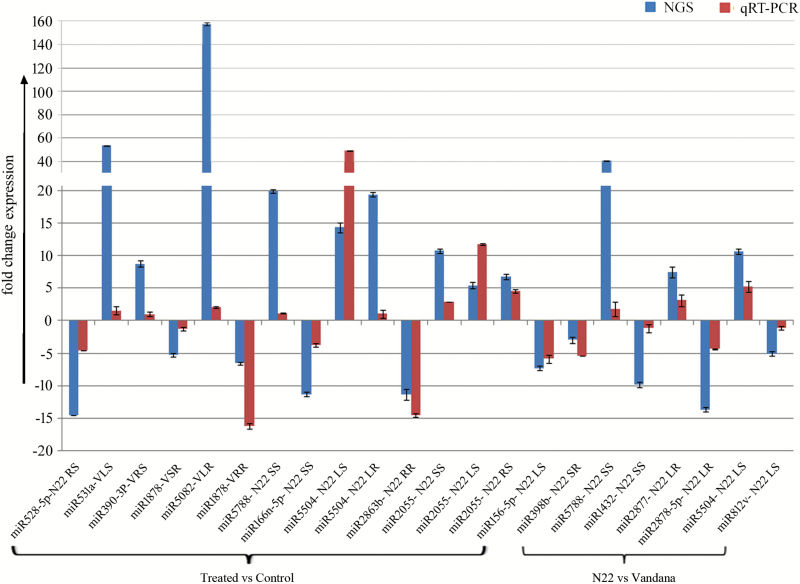
Expression validation of miRNAs through qRT-PCR. The differential expression of miRNAs was validated using 17 miRNAs in 22 different comparisons. The fold change expression of miRNAs was calculated from NGS data and qPCR experiment to compare their expression. The *y*-axis shows the fold change expression of miRNAs. Bars represent the mean±SE of three biological replicates. (This figure is available in color at *JXB* online.)

**Fig. 7.**
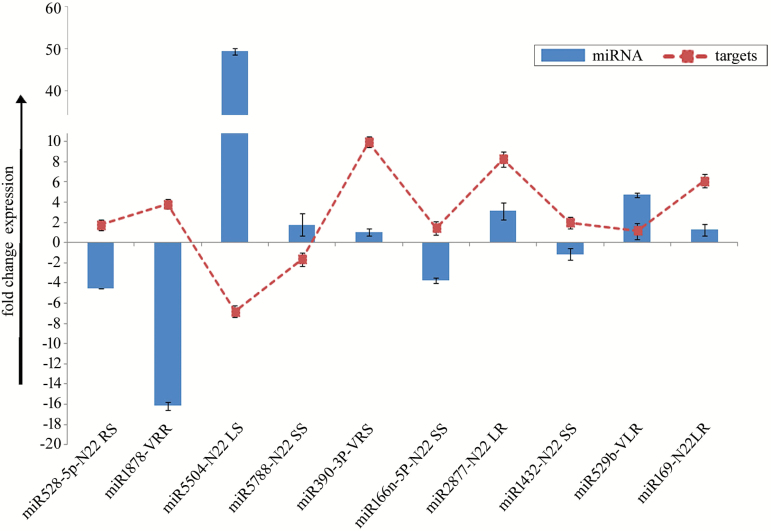
Expression correlation of miRNAs and their target genes. The *y*-axis shows the fold change expression of miRNAs and target genes in stressed sample in comparison with their respective control sample. Bars represent the mean±SE of three biological replicates. (This figure is available in color at *JXB* online.)

In order to validate the deep sequencing data, expression analysis of 17 miRNAs was performed in 22 different combinations of heat treatment *versus* control and N22 *versus* Vandana (Fig. 6). The results showed a similar trend of expression of miRNAs in NGS and qPCR, though the degree of expression varied. Further, expression correlation of miRNAs and their target genes was analysed and suggested a negative correlation between miRNAs and gene targets (Fig. 7).

### Function analysis

For each library, target transcripts representing genes with a known function were categorized into biological process, cellular component and molecular function according to the ontological definitions of the Gene Ontology (GO) terms (Supplementary Table S8). The target transcripts of miRNAs in the molecular function category were related to binding, protein binding, catalytic activity, hydrolase, kinase, transferase, nuclease, transporter, signal transducer, enzyme regulator activity, motor activity, receptor activity, and structural molecule activity. Most of the miRNA target genes were assigned to the binding category whose present sequences appear to be involved in nucleic acid binding, protein binding and ion binding. Since these target sequences encode transcription factors, it is in accordance with previous reports that a large proportion of the miRNA targets encode transcription factors.

The number of miRNA targets falling under different categories of molecular function was also analysed for different libraries. Significant difference was not observed in the shoot library of Vandana. However, root tissue of Vandana showed a distinct difference in the number of miRNA targets falling under catalytic activity, hydrolase activity, kinase activity, nucleotide binding, protein binding, and RNA binding. In particular, 11 miRNA targets showed kinase activity in control and stress root tissue and only three in recovery root tissue. Similarly, 12, 13, and 5 miRNA targets were identified to play a role in nucleotide binding in control, stress, and recovery root tissue, respectively. Similar to Vandana, shoot libraries of N22 also did not show much difference in number of miRNA targets under different molecular function categories; however, a significant difference was observed in root of N22. A distinct difference in root libraries was observed in the number of miRNA targets associated with binding, catalytic activity, hydrolase activity, kinase activity, nucleotide binding, and protein binding; 10, 13, and 9 miRNA targets showed kinase activity in control, stress, and recovery root of N22, respectively.

In the biological processes category, the total number of miRNA targets in each library fell into multiple classes. However, it is notable that some of these transcripts in the biological process category were related to stress response. In the shoot library of Vandana, difference in miRNA targets falling in different categories was not significant when compared among control, stress, and recovery. However, the difference was more evident in root tissue of Vandana. Here, 23, 24, and 15 target transcripts were found in control, stress, and recovery tissue, respectively. A significant difference was also observed in miRNA targets associated with biosynthetic processes, metabolic processes, protein modification processes, signal transduction, and transport. In particular, the target transcripts associated with protein modification processes were 14, 13, and 4, while metabolic processes showed 30, 25, and 19 transcripts in control, stress, and recovery root, respectively.

Similar to Vandana, shoot tissue of N22 did not show much variation in terms of number of miRNA targets falling under different categories of biological processes. However, root tissue of N22 showed significant variation. Control, stress, and recovery root tissue showed, respectively, 27, 37, and 24 target transcripts under biological processes; 23, 27, and 17 target transcripts under biosynthetic processes; 33, 37, and 27 target transcripts under cellular processes; 30, 37, and 27 target transcripts under metabolic processes; 12, 16, and 11 target transcripts under protein modification processes; 12, 20, and 12 target transcripts under stress response; 4, 8, and 4 target transcripts under response to endogenous stimuli; and 9, 11, and 7 target transcripts under signal transduction.

Cellular components of miRNA targets were also analysed for control, stress, and recovery libraries of N22 and Vandana. In terms of number of miRNA targets showing different cellular locations, Vandana shoot tissue did not show much variation in different libraries. However, root tissue showed variation under cellular component: cytosol, intracellular, membrane, mitochondrion, nucleus, and plasma membrane. In particular transcripts localizing in plasma membrane were 11 in control, 12 in stress and 5 in recovery of Vandana root. N22 root showed 13, 15, and 10 transcripts localizing in plasma membrane in control, stress, and recovery, respectively. In N22 shoot, a significant difference in miRNA targets localizing in nucleus was observed. The number of transcripts localizing in nucleus was 16 (control), 11 (stress), and 18 (recovery). In general, target genes localizing in different cellular components were lesser in stress as compared with control. In comparison with shoot, root tissue of N22 showed a higher number of transcripts localizing in different cellular components under stress condition (Supplementary Table S8).

## Discussion

This study is specifically important in addressing three major issues of heat stress response which have not been previously reported in miRNA/transcriptome studies on high temperature stress. First, we have studied the miRNA regulation during short- as well as long-duration heat stress. Second, while treating the plants with high temperature, the day/night difference of temperature was maintained. Third, microRNA regulation after recovery was also studied.

In all, 162 miRNA families were identified in two rice genotypes, which included monocot preferential miRNA families-miR437, miR444, miR528, miR1318, miR1432, and miR1436 (Zhang *et al.*, 2011; Pandey *et al.*, 2014). Similar to other reports of deep sequencing of microRNAs in rice, the expression of conserved miRNA families was significantly higher than that of non-conserved miRNAs (Sunkar *et al.*, 2008; Zhu *et al.*, 2008; Li *et al.*, 2011; Chen *et al.*, 2013). MicroRNA166 was most abundantly present across the 16 rice libraries sequenced in this study. Based on this study, we classified miRNA families into preferential expression in cultivars (N22 and Vandana), tissue (shoot and root), and treatments (control and heat stressed). However, this classification needs to be further validated as small RNA sequencing may not be saturated to cover all the small RNAs in a given sample. The majority of miRNA families were common. N22 preferential miRNA families included osa-miR1427, osa-miR1848, osa-miR1865, osa-miR2872, osa-miR5155, and osa-miR5484, while Vandana preferential miRNA families were osa-miR414 and osa-miR531. These miRNAs were suggested as potential candidates for selection in domestication, novel in cultivated rice when compared with wild rice (Wang *et al.*, 2012). In addition, microRNA families osa-miR2120, osa-miR1846, osa-miR5079, osa-miR1318, osa-miR1863, osa-miR2275, osa-miR5072, osa-miR5160, osa-miR5162, osa-miR5505, and osa-miR5513 were suggested to be involved in cultivated rice domestication and were present in both Vandana and N22. While a previous study by Wang *et al.*, (2012) was carried out with wild rice (*Oryza rufipogon*) to determine the role of miRNA genes in rice domestication, this study supports those findings for miRNAs identified in cultivated rice (*Oryza sativa*). *O. sativa* has been arranged into five distinct groups, corresponding to indica, aus, aromatic, temperate japonica, and tropical japonica rices (Garris *et al.*, 2005), and this study provided an opportunity to investigate miRNAs of the indica (Vandana) and aus (N22) groups.

The miRNA families showing preferential expression in root included osa-miR3981, which targets indole-3-glycerol phosphate lyase, catalysing indole-3-glycerol phosphate into indole and glyceraldehyde-3-phosphate. Indole is used for biosynthesis of the C-7-methoxy derivative of 2,4-dihydroxy-2*H*-1,4-benzoxazin-3(4*H*)-one (DIBOA), known as DIMBOA, a predominant benzoxazinoid found in roots (Rice *et al.*, 2005; Dick *et al.*, 2012). Another such miRNA family, osa-miR2876, targets splicing factor U2AF. Expression analysis of Arabidopsis U2AF showed strong expression of AtU2AF35b in whole young roots and root tips and AtU2AF35a in root vascular regions (Wang and Brendel, 2006). The miRNA families showing preferential expression in shoot included osa-miR1439, which targets no apical meristem protein involved in control of boundary formation and lateral organ separation, which is critical for proper leaf and flower patterning (Cheng *et al.*, 2012). Another such, miRNA172, regulates AP2 domain-containing protein, which regulates the development of shoot meristems (Licausi *et al.*, 2013). osa-miR1864 targets ternary complex factor MIP1, which interacts with MADS-box TF involved in meristem determination during floral transition (Causier *et al.*, 2003).

Expression of osa-miR1436 was recorded in both genotypes but preferentially in stressed tissues. It was found putatively to target a large number of genes such as CRAL/TRIO domain-containing protein, scramblase, transmembrane protein, tyrosine protein kinase domain-containing protein, glycoprotein 3-α-L-fucosyltransferase A, cytochrome P450, serine/threonine-protein kinase, WRKY34, etc., suggesting its general response to stress. This miRNA was shown to be associated with arsenic stress response in *Brassica juncea* (Srivastava *et al.*, 2013). The expression of osa-miR1436 was decreased while the expression of its target gene was increased upon glyphosate treatments (Unver *et al.*, 2010). Sunkar *et al.*, (2008) suggested that miR1436 may be involved in drought stress in rice. Osa-miR5076 was also identified preferentially in the stressed library of both the genotypes. The predicted target of osa-miR5076 is ferredoxin-dependent glutamate synthase involved in nitrogen assimilation in rice. High temperature affects nitrogen metabolism of plants significantly (Xu and Zhou, 2006).

The miRNAs expression profile was further evaluated with a primary focus on the heat-tolerant N22 genotype of rice. In comparison with control, the SDS shoot of N22 showed up-regulation of osa-miR5788, osa-miR1427, and osa-miR2055, and down-regulation of osa-miR166n-5p, osa-miR1425, and osa-miR399. The target of osa-miR5788 is U-box domain containing heat shock protein. Overexpression of AtCHIP, a U-box protein, in Arabidopsis rendered plants more sensitive to both low- and high-temperature treatments (Yan *et al.*, 2003). miR399 controls phosphorus homeostasis by regulating the expression of a ubiquitin-conjugating E2 enzyme in Arabidopsis (Chiou *et al.*, 2006) and participates in the regulation of multiple nutrient starvation responses (Hu *et al.*, 2015).

The up-regulated microRNAs in N22 shoot after LDS (in comparison with control) were osa-miR5083, osa-miR5504, osa-miR5160, osa-miR5072, osa-miR2055, and osa-miR1427, and the down-regulated miRNAs were osa-miR166, osa-miR5073, osa-miR156, and osa-miR390. The miR156 targets squamosa promoter binding protein-like (SPL) and dihydroflavonol-4-reductase (DFR) pathways to coordinate the relationship between development and abiotic stress tolerance in plants (Cui *et al.*, 2014). Recently it was shown that miR156 isoforms are highly induced after heat stress and are functionally important for heat stress memory. miR156 promotes sustained expression of stress-responsive genes and is critical after high temperature stress adaptation to recurring heat stress in Arabidopsis (Stief *et al.*, 2014). This was not differentially expressed in SDS but appeared preferentially in LDS. miR390 was down-regulated during cadmium stress in rice (Ding *et al.*, 2011). It contributes to coordinate multiple phytohormone signals for the regulation of cell growth and senescence in plants (Curaba *et al.*, 2014). This miRNA targets STRUBBELIG-RECEPTOR FAMILY (SRF) 6 precursor, which was strongly induced in plants exposed for a prolonged time to heat stress (Eyüboglu *et al.*, 2007). Our results indicate that miR156 and miR390 may be involved in acclimation or adaptation to long duration heat stress in rice, which needs to be confirmed in further studies.

The up-regulated miRNAs in shoot of N22 after REC (in comparison with control) were osa-miR1863a, osa-miR1427, osa-miR2055, osa-miR168a-3p, osa-miR167a-3p, osa-miR5083, osa-miR5160, and osa-miR5072. miR1863 is required for silencing heterochromatin by methylation in rice (Wu *et al.*, 2010). Recent studies show the involvement of the DNA methylation process in high temperature response in plants (Sanchez and Paszkowski, 2014; Naydenov *et al.*, 2015). Imprinting of genes may help in recovery of stressed plants after a heat stress episode. osa-miR168a-3p was shown to be an arsenic-responsive miRNA in the shoots of rice (Yu *et al.*, 2012). Further, miR168a-3p and miR167a-3p were identified as heat stress (37 °C for 6 h)-responsive microRNAs in Arabidopsis (Barciszewska-Pacak *et al.*, 2015). osa-miR5083, osa-miR5160, and osa-miR5072 were up-regulated after LDS and REC indicating their significant role in response and recovery from long duration high temperature stress in rice. It is interesting to note that osa-miR2055 and osa-miR1427 were up-regulated after SDS, LDS, and REC of N22 suggesting a common function of these miRNAs in regulating tolerance to high temperature. The miRNAs showing down-regulation after REC were osa-miR528-5p, osa-miR528, osa-miR166, osa-miR5073, osa-miR2863, osa-miR6246, and osa-miR1863c. miR528 was down-regulated in rice seedlings treated with hydrogen peroxide (H_2_O_2_) to induce oxidative stress (Li *et al.*, 2011). osa-miR166 was down-regulated after SDS, LDS, and REC while osa-miR5073 showed down-regulation only after LDS and REC of N22 shoot indicating some miRNAs are recruited only when the duration of heat stress is long, and they continue their role during recovery too.

In comparison with control, the root tissue of N22 showed up-regulation of osa-miR5504, osa-miR169, osa-miR1427, osa-miR390, osa-miR166b-5p, osa-miR5082, and osa-miR319a-3p after SDS. Interestingly, osa-miR1427 showed up-regulation in shoot also after SDS, LDS, and REC. In a recent study, osa-miR169i-5p.2, osa-miR390-3p, and osa-miR5082 showed high abundance in root tips suggesting their important role in root development of rice (Ma *et al.*, 2013). The up-regulation of osa-miR169, osa-miR390, and osa-miR5082 in N22 root after SDS suggests that these miRNAs may be involved in root development of N22 under high temperature stress. osa-miR319 was suggested to be an oxidative stress-responsive miRNA family in rice (Li *et al.*, 2011). We observed the down-regulation of osa-miR2275d and osa-miR1850.1 in N22 root after SDS. osa-miR2275 was shown to be regulated by drought and cold stress in rice (Barrera-Figueroa *et al.*, 2012). It is important to note that osa-miR2275 is involved in regulation of biogenesis of secondary small interfering RNAs (siRNAs) of either 21 or 24 nucleotides in a phased manner (Song *et al.*, 2012), which may have a significant function in abiotic stress response.

The significantly up-regulated microRNAs in N22 root after LDS (in comparison with control) were osa-miR5504, osa-miR1427, osa-miR5083, osa-miR169e, osa-miR156, and osa-miR6250. The induced expression of osa-miR5504 and osa-miR1427 was observed in roots after SDS also. miR6250 showed high abundance in rice roots in response to arsenite treatment (Liu, 2012). Significantly down-regulated miRNAs were osa-miR169a, osa-miR1863, osa-miR2878, osa-miR1425, osa-miR1876, osa-miR171, osa-miR1874, and osa-miR159. Interestingly, osa-miR169e was up-regulated while osa-miR169a was down-regulated in root of N22 after LDS. Tissue differential expression of members in the same family of miRNAs has been reported previously (Jeong *et al.*, 2011). Numerous miRNAs were down-regulated in root tissue of N22 after REC suggesting the up-regulation of target genes. Expression analysis of 13 genes and 9 miRNAs suggested root as more responsive in N22 after recovery from heat stress treatments (Sailaja *et al.*, 2014).

The miRNA composition and expression pattern in the heat susceptible cultivar Vandana was largely different form heat tolerant N22 when analysed after SDS, LDS, and REC (Fig. 4, and Tables S4 and S5). The miRNAs expressed preferentially in the tolerant rice genotype N22 at high temperature were osa-miR1439, osa-miR1848, osa-miR2096, osa-miR2106, osa-miR2875, osa-miR3981, osa-miR5079, osa-miR5151, osa-miR5484, osa-miR5792, and osa-miR5812. These miRNAs may have a more specific role in heat stress tolerance. MicroRNA1439 was suggested to be involved in the homeostasis of metal ions and plays a role in plant response to salt stress (Wu *et al.*, 2012). Recently, osa-miR1848 was shown to target the obtusifoliol 14α-demethylase gene (*OsCYP51G3*) and mediates the biosynthesis of phytosterols and brassinosteroids during development and stress response (Xia *et al.*, 2015). osa-miR2106 was found to be up-regulated in arsenic stress in rice (Yu *et al.*, 2012). Beside the specific miRNAs expressed preferentially in heat tolerant N22 as discussed above, there were a number of miRNAs that showed differential expression when compared with Vandana. Higher expression of osa-miR1859, osa-miR5504, and osa-miR5513 was observed in N22 than in Vandana shoot after SDS, LDS, and REC. osa-miR408-3p was down-regulated after SDS, LDS, and REC of N22 shoot when compared with corresponding samples of Vandana shoot. Interestingly, osa-miR408 was shown to be up-regulated in N22 and Vandana during drought stress in young seedlings, as well as in flag leaf and spikelets of adult plants (Mutum *et al.*, 2013). Both these genotypes are drought tolerant, however, contradicting their response to high temperature. It would be interesting to analyse the differential role of the osa-miR408 family in drought and heat stress response in rice. miR408 is suggested to target the plastocyanin-like protein family and helps in maintaining the cellular redox state (Mutum *et al.*, 2013).

It was interesting to observe that a larger number of miRNAs were up-regulated in N22 SR than in Vandana SR suggesting that root was more responsive in N22. We had made a similar observation in an earlier study with a limited number of miRNAs and transcripts (Sailaja *et al.*, 2014). The role of osa-miR5150-3p, osa-miR528-5p, osa-miR398b, and osa-miR408-3p in recovery response of the heat tolerant genotype needs to be further characterized as these miRNAs showed differential expression in N22 RR when compared with Vandana RR. Overall, this study suggests that N22 has distinct miRNA-mediated gene regulation machinery in comparison with Vandana after various heat stress treatments.

The functional analysis of target transcripts of miRNAs identified in different rice libraries suggests that root tissue is more responsive under heat stress treatments and recovery. The molecular function and biological process category of miRNA targets of N22 and Vandana showed significant difference in various GO categories after SDS, LDS, and REC in root, while shoot did not show much difference. Further, functional analysis of target transcripts suggested that N22 root has a more efficient recovery process as compared with Vandana. Our previous study based on expression analysis of a set of genes and miRNAs indicated that root is more responsive than shoot during heat stress in rice and N22 has a more efficient recovery mechanism to cope with high temperature stress than Vandana (Sailaja *et al.*, 2014).

In conclusion, we identified miRNAs from 16 rice samples, their target genes and molecular pathways that are regulated by elevated temperature in rice. This is the first report of whole-genome miRNAs expression at a wider scale that includes profiling of miRNA expressed in root and shoot of heat tolerant and susceptible rice cultivars during control, short and long durations of heat stress treatment and also recovery after LDS. Further, unlike other reports where heat stress was given for a certain duration, generally a few hours, and then returned to optimal temperature, we attempted to map the miRNA expression by maintaining the day/night temperature difference in control and elevated temperature treatments, which is a more realistic situation. These results have opened a baseline for understanding miRNA-mediated regulation of rice response to high temperature. Even though we see contrasting response to heat stress in shoot growth of N22 and Vandana, the miRNA expression shows more differential activity in response to heat in root than in shoot. This study suggests that heat susceptible and tolerant rice cultivars have a different landscape of miRNA expression and regulation at elevated temperature, which might be associated with the molecular events leading to contrasting responses of rice genotypes to heat stress. The study provides the first comprehensive and specific miRNA profile of heat-induced miRNAs in shoot and root of rice. The identification of genotype and tissue preferential miRNAs will help in widening the knowledge base of miRNA-mediated gene regulation in rice.

## Supplementary data

Supplementary data are available at JXB online.

Fig. S1. The length distribution of small RNA reads.

Table S1. List of miRNAs and target gene primers used in this study.

Table S2. List of miRNA families identified in different rice libraries.

Table S3. Predicted target genes of miRNAs expressed preferentially in control or stressed tissue of N22 and Vandana.

Table S4. Mature miRNAs along with details of read sequence and read count.

Table S5. List of differentially expressed miRNAs in 36 pairwise combinations.

Table S6. List of candidate target genes of differentially expressed miRNAs.

Table S7. List of miRNAs showing expression preferentially in one tissue or treatment and absent in others.

Table S8. Categorization of miRNA target transcripts into biological process, cellular component and molecular function according to the ontological definitions of the GO terms.

## Supplementary Material

supplementary_figure_S1

supplementary_table_S1

supplementary_table_S2

supplementary_table_S3

supplementary_table_S4

supplementary_table_S5

supplementary_table_S6

supplementary_table_S7

supplementary_table_S8
