# Supplementary material for: Genome-wide changes in microRNA expression during short and prolonged heat stress and recovery in contrasting rice cultivars
Source: J Exp Bot. 2017 Apr 12;68(9):2399–412. doi: 10.1093/jxb/erx111 (PMC5447883; doi:10.1093/jxb/erx111)
Supplement: supplementary_figure_S1 [file erx111_suppl_supplementary_figure_S1.pdf]

Vandana-CS

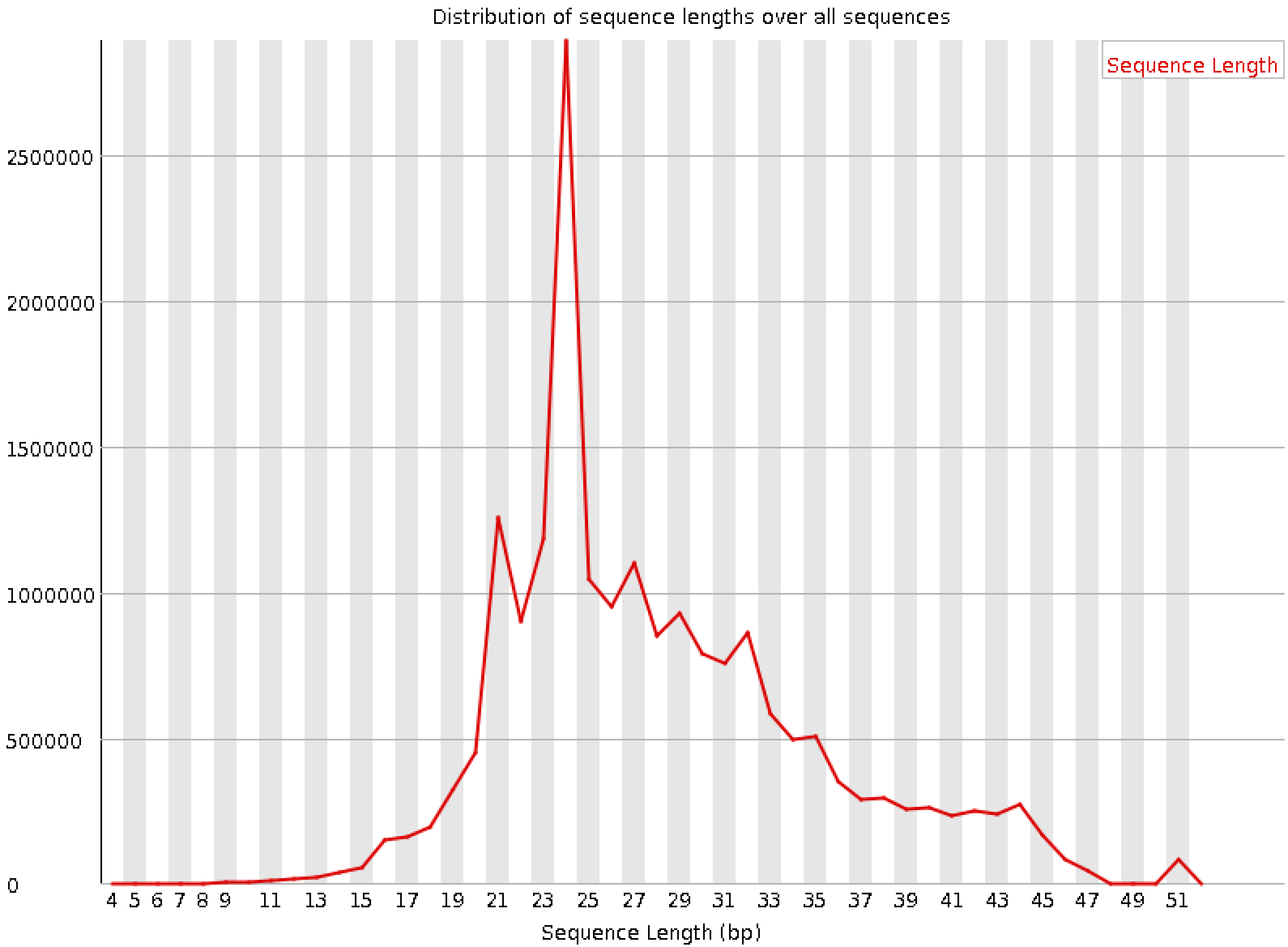
Distribution of sequence lengths over all sequences
Sequence Length
2500000
2000000
1500000
1000000
500000
0
4 5 6 7 8 9 11 13 15 17 19 21 23 25 27 29 31 33 35 37 39 41 43 45 47 49 51
Sequence Length (bp)

Vandana-CR

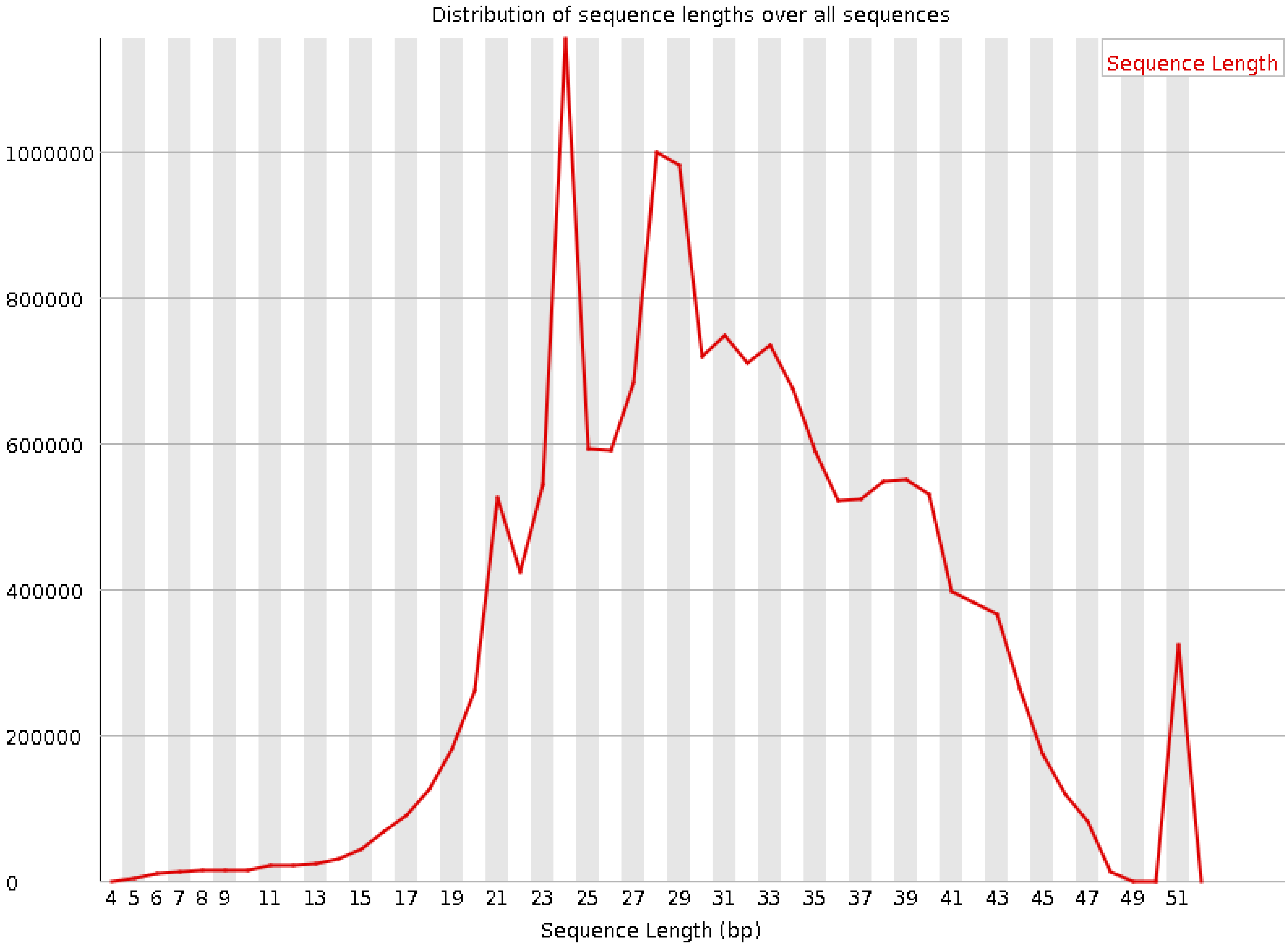

Distribution of sequence lengths over all sequences
Sequence Length
1000000
800000
600000
400000
200000
0
4 5 6 7 8 9 11 13 15 17 19 21 23 25 27 29 31 33 35 37 39 41 43 45 47 49 51
Sequence Length (bp)

Vandana-LS

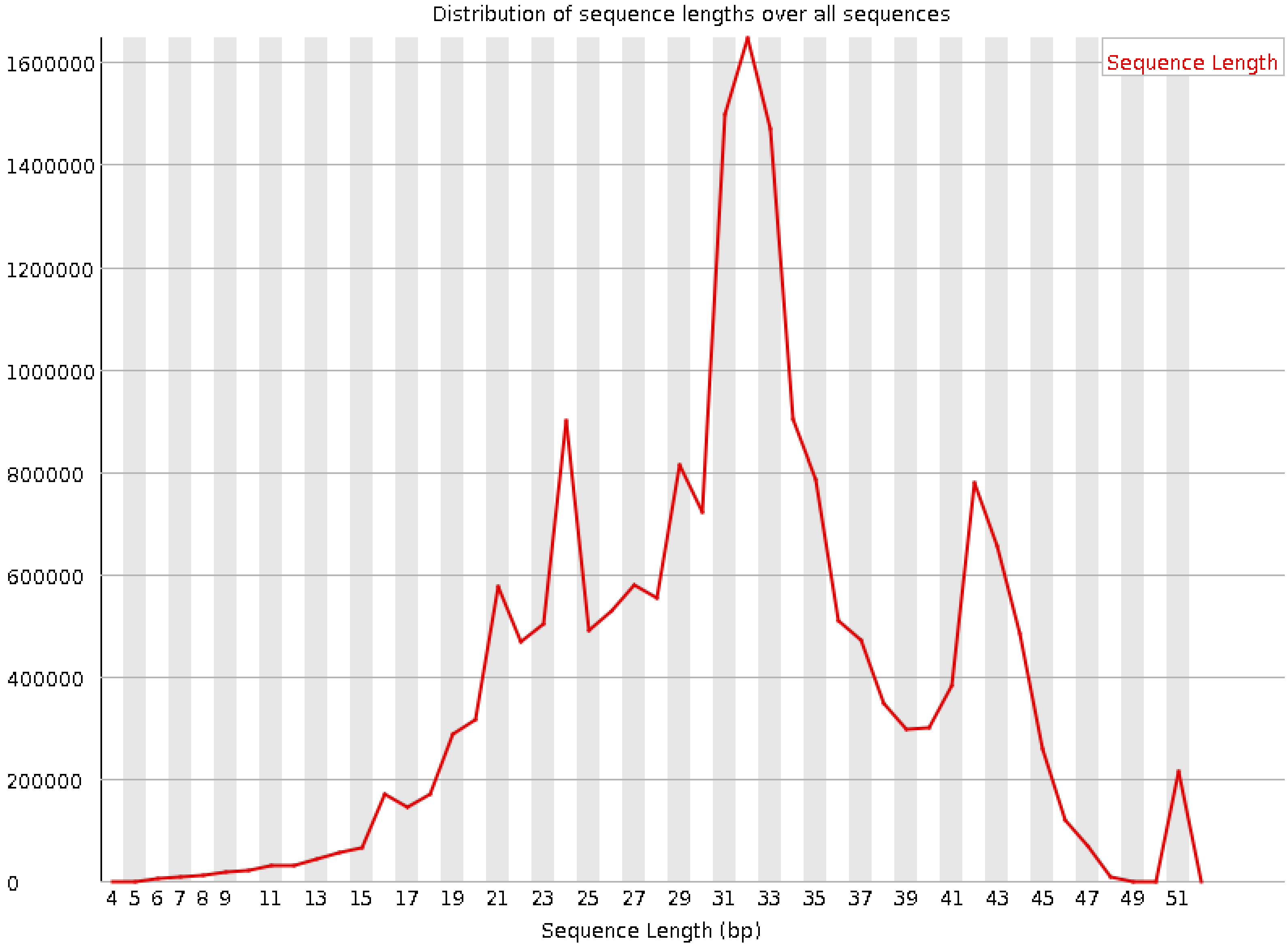
Distribution of sequence lengths over all sequences
Sequence Length
1600000
1400000
1200000
1000000
800000
600000
400000
200000
0
4 5 6 7 8 9 11 13 15 17 19 21 23 25 27 29 31 33 35 37 39 41 43 45 47 49 51
Sequence Length (bp)

Vandana-LR

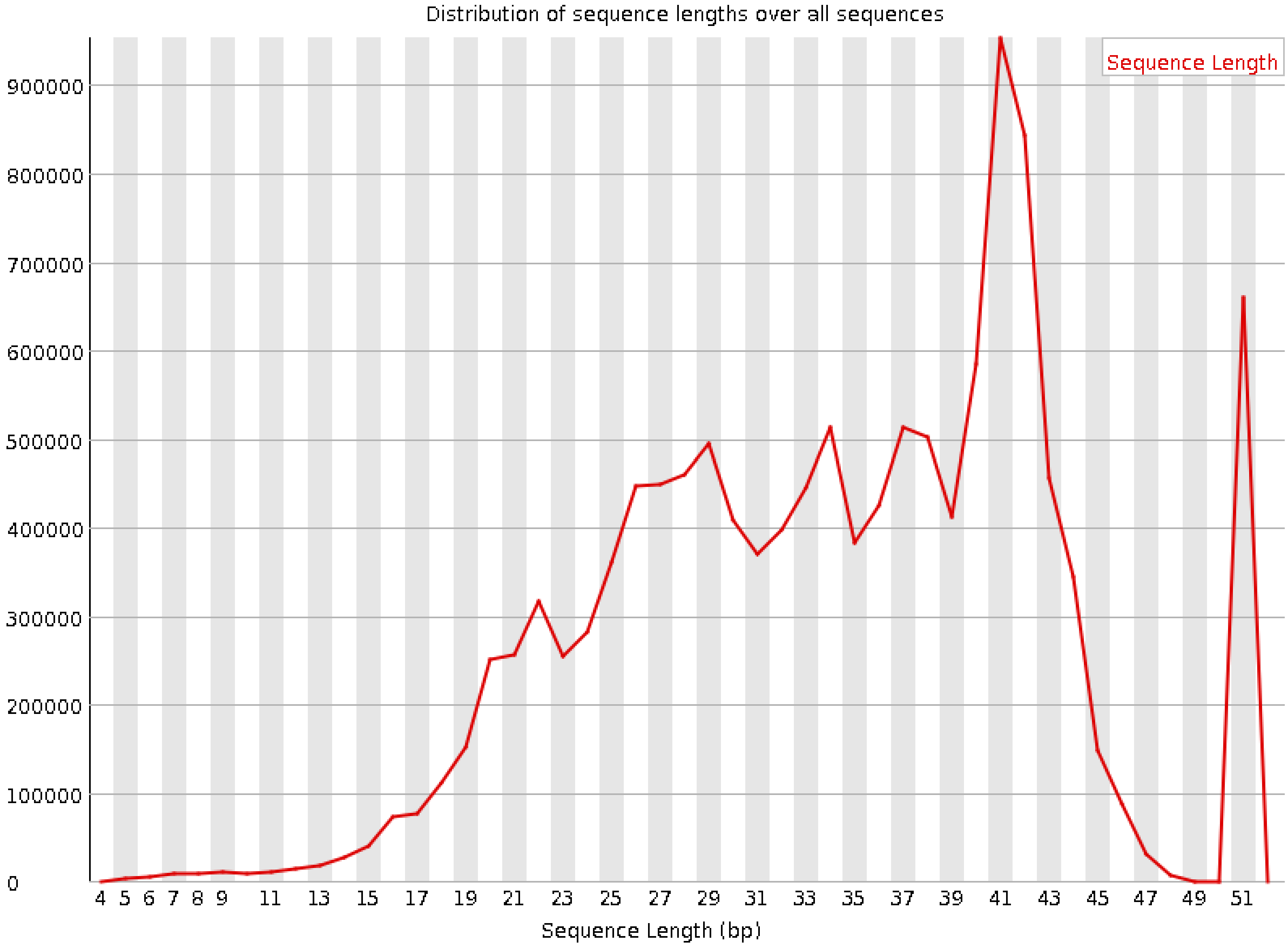

Distribution of sequence lengths over all sequences
Sequence Length
900000
800000
700000
600000
500000
400000
300000
200000
100000
0
4 5 6 7 8 9 11 13 15 17 19 21 23 25 27 29 31 33 35 37 39 41 43 45 47 49 51
Sequence Length (bp)

Vandana-SS

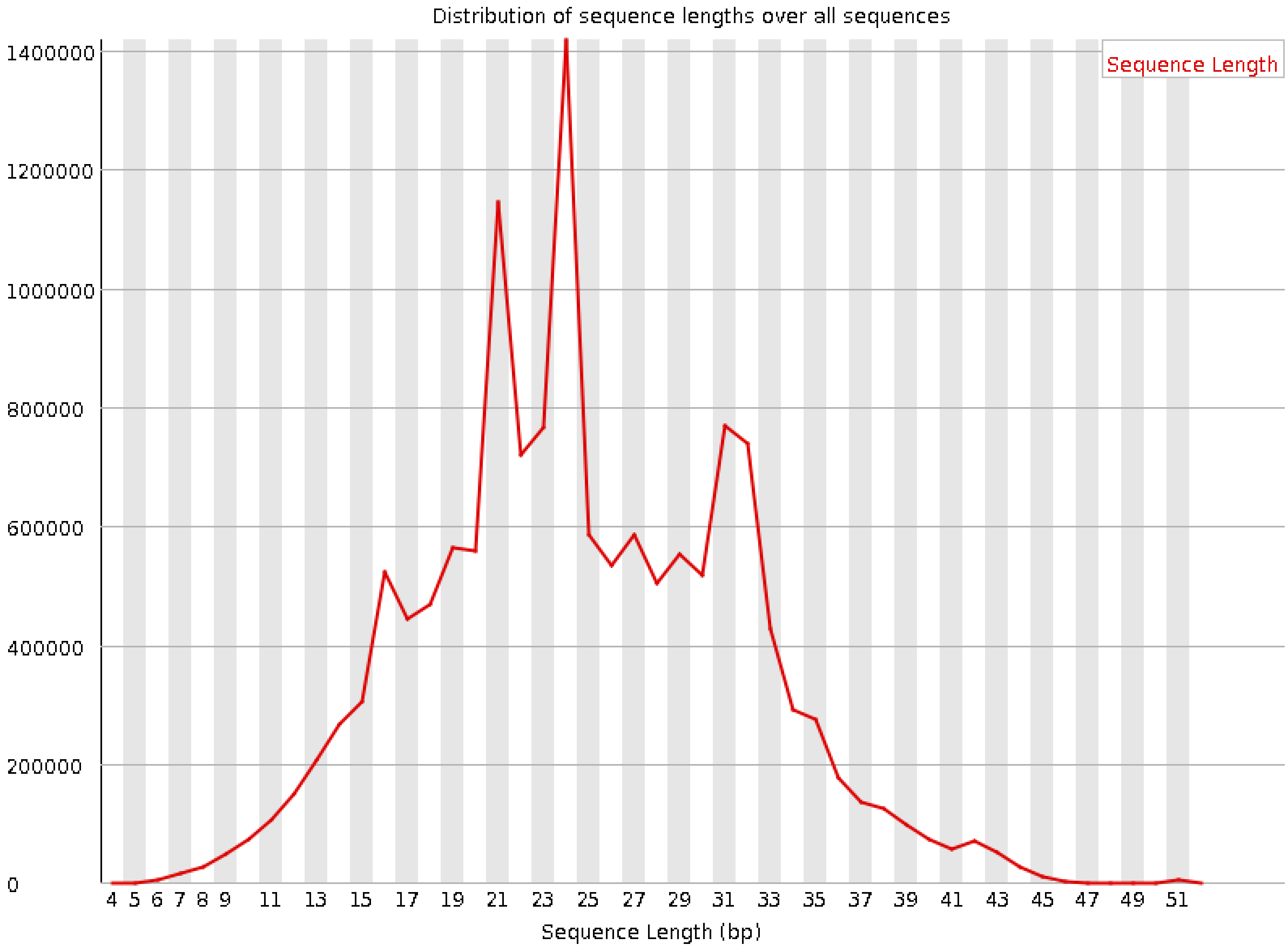

Distribution of sequence lengths over all sequences
Sequence Length
1400000
1200000
1000000
800000
600000
400000
200000
0
4 5 6 7 8 9 11 13 15 17 19 21 23 25 27 29 31 33 35 37 39 41 43 45 47 49 51
Sequence Length (bp)

Vandana-SR

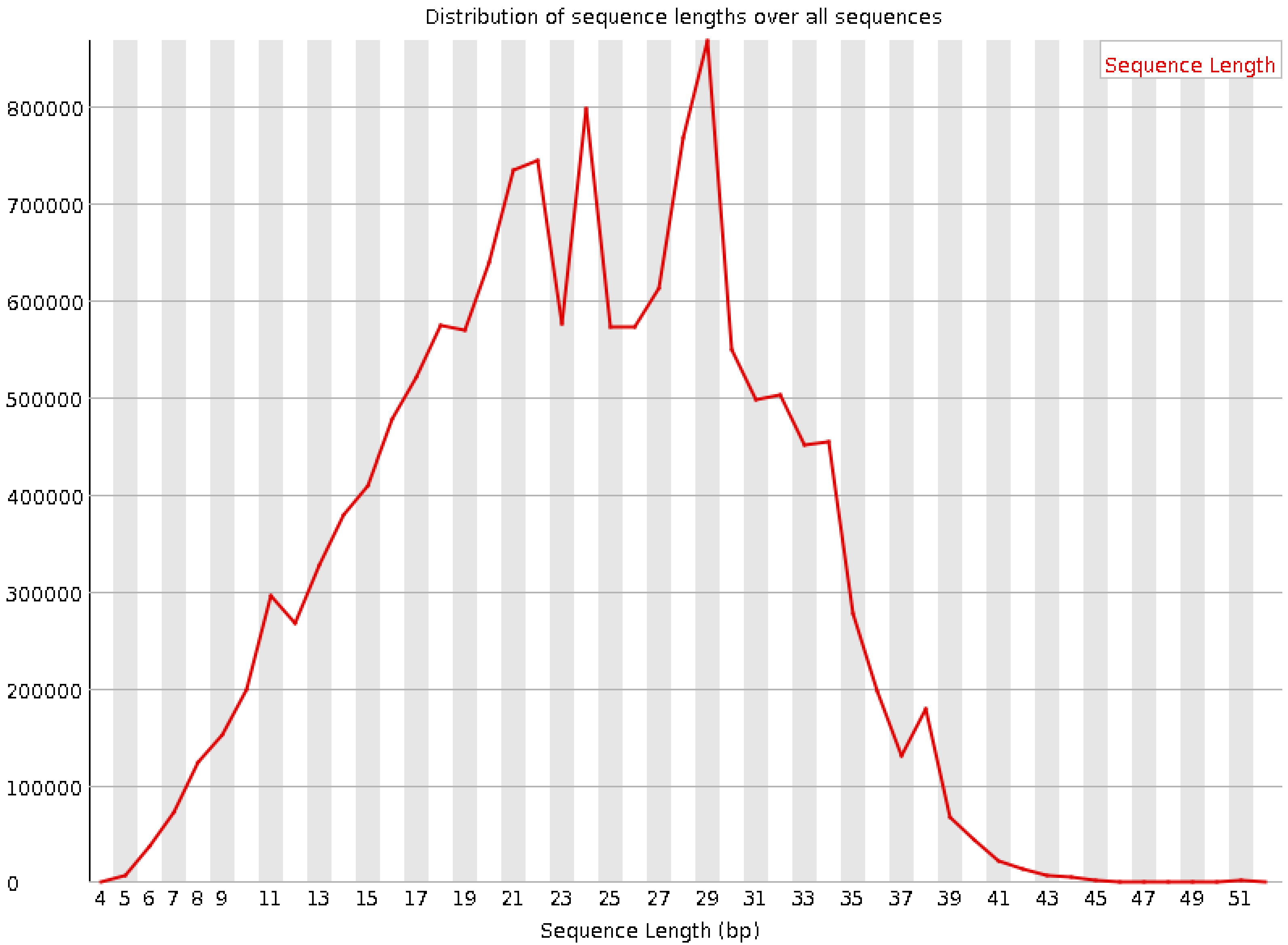
Distribution of sequence lengths over all sequences
Sequence Length
800000
700000
600000
500000
400000
300000
200000
100000
0
4 5 6 7 8 9 11 13 15 17 19 21 23 25 27 29 31 33 35 37 39 41 43 45 47 49 51
Sequence Length (bp)

Vandana-RS

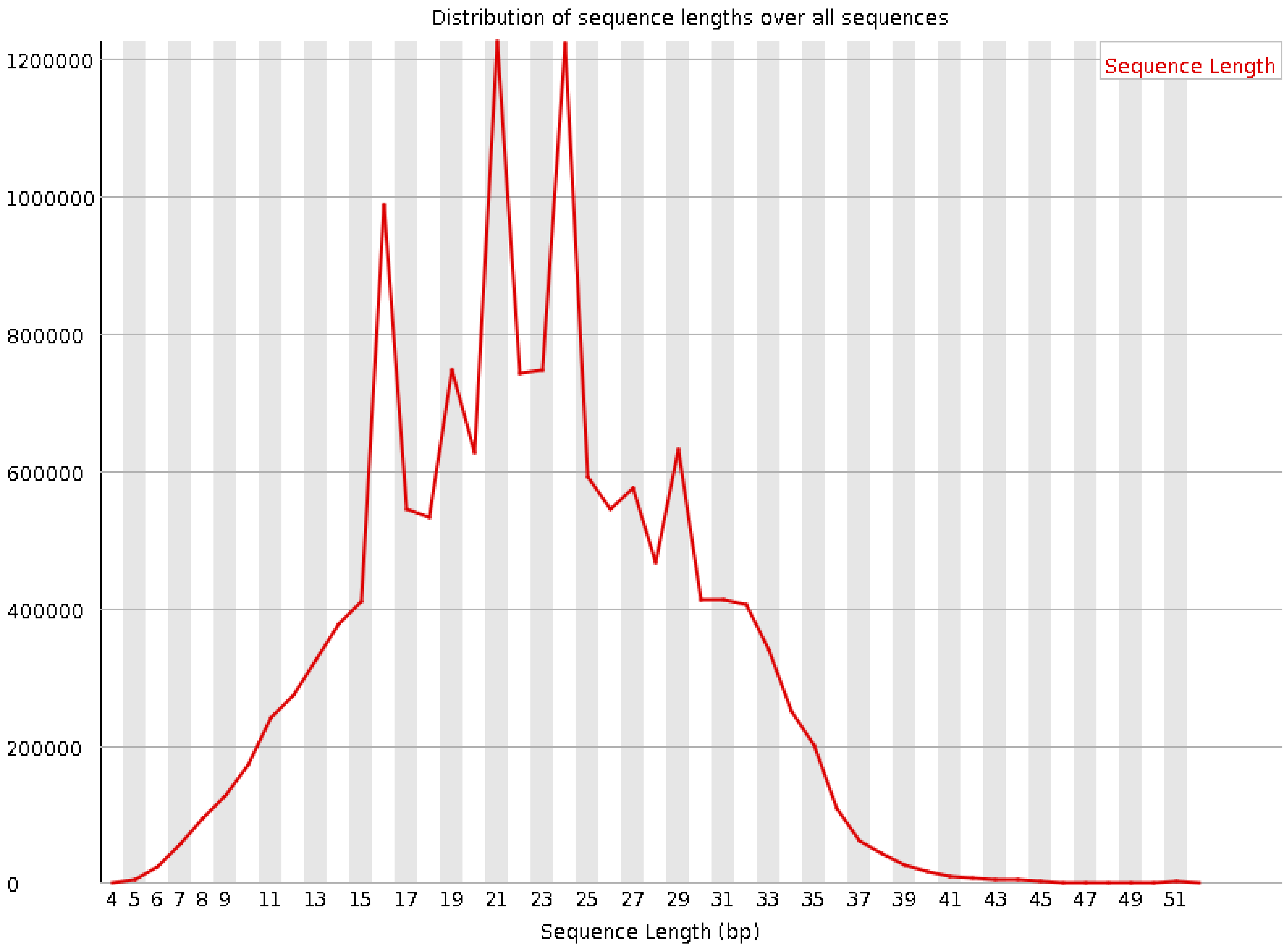

Distribution of sequence lengths over all sequences
Sequence Length
1200000
1000000
800000
600000
400000
200000
0
4 5 6 7 8 9 11 13 15 17 19 21 23 25 27 29 31 33 35 37 39 41 43 45 47 49 51
Sequence Length (bp)

Vandana-RR

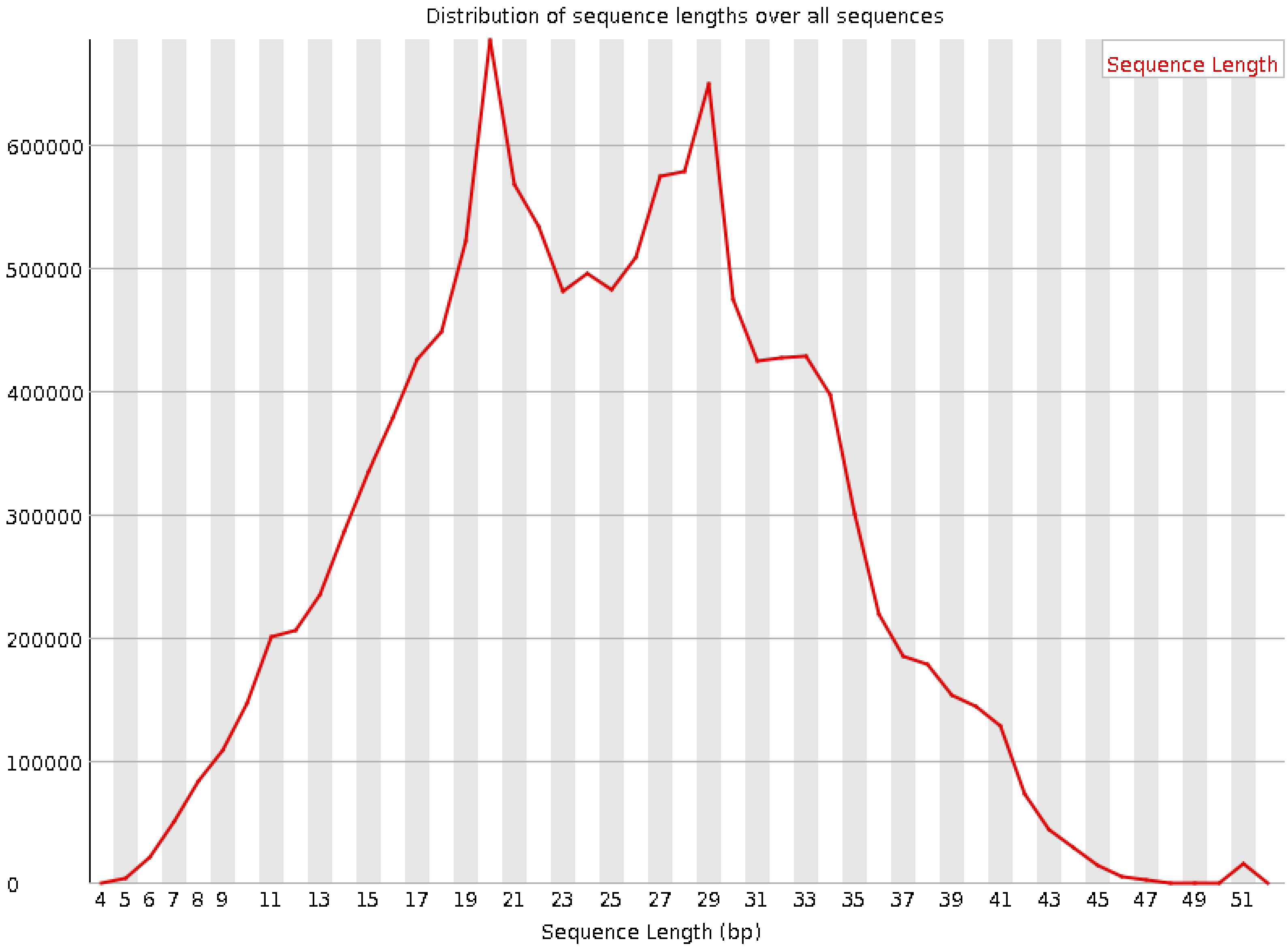
Distribution of sequence lengths over all sequences
Sequence Length
600000
500000
400000
300000
200000
100000
0
4 5 6 7 8 9 11 13 15 17 19 21 23 25 27 29 31 33 35 37 39 41 43 45 47 49 51
Sequence Length (bp)

N22-CS

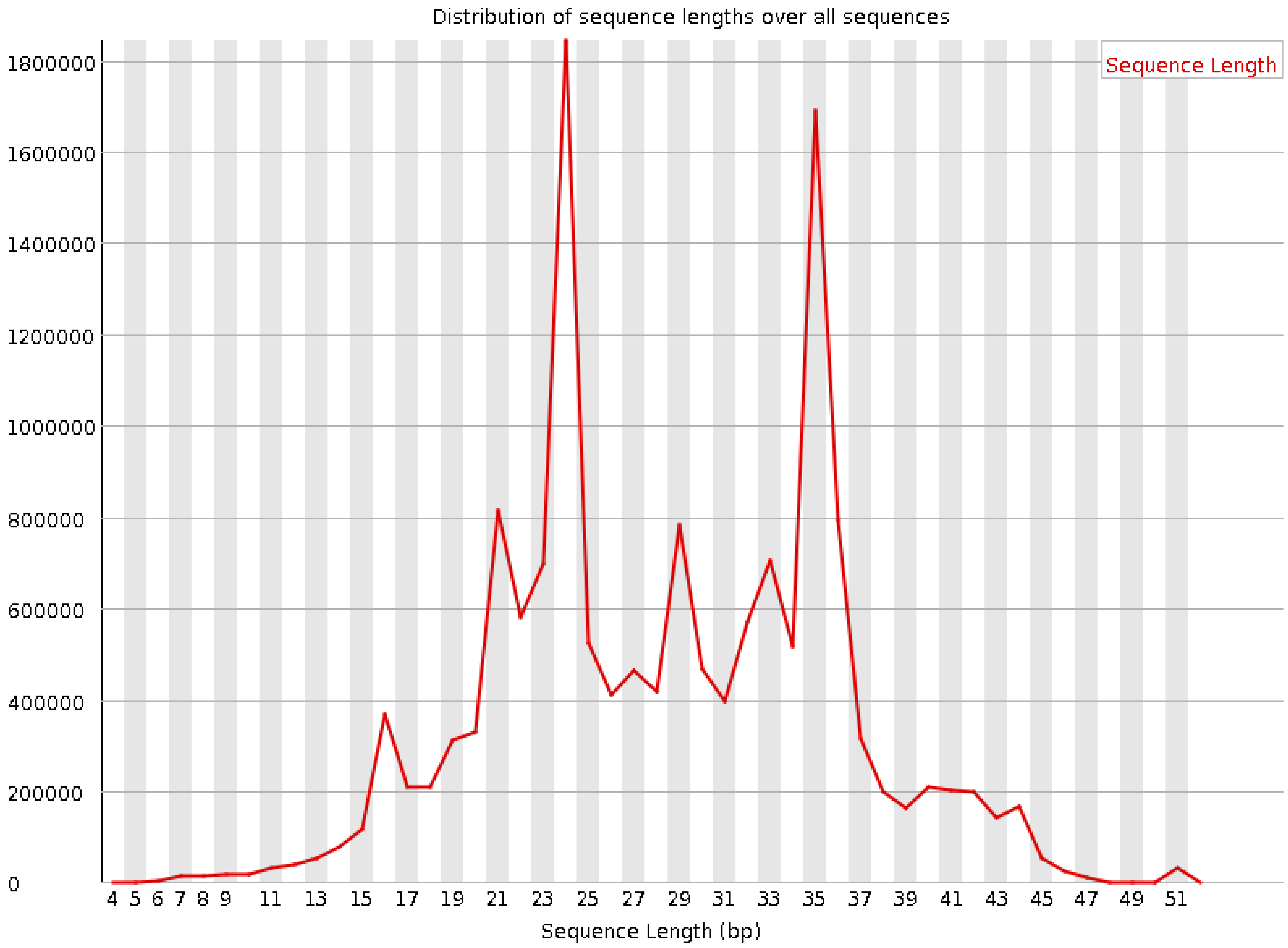
Distribution of sequence lengths over all sequences
Sequence Length
1800000
1600000
1400000
1200000
1000000
800000
600000
400000
200000
0
4 5 6 7 8 9 11 13 15 17 19 21 23 25 27 29 31 33 35 37 39 41 43 45 47 49 51
Sequence Length (bp)

N22-CR

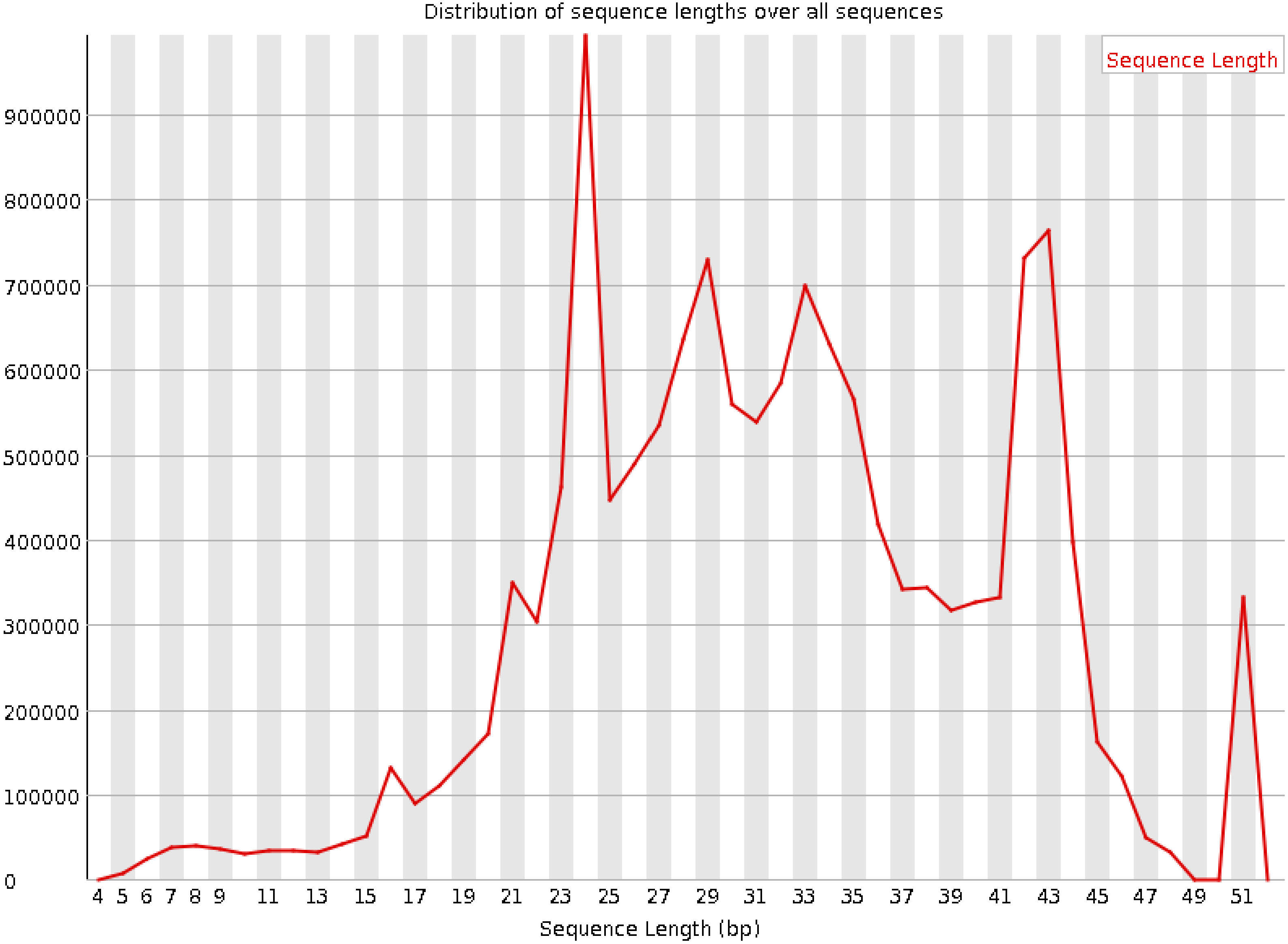

Distribution of sequence lengths over all sequences
Sequence Length
900000
800000
700000
600000
500000
400000
300000
200000
100000
0
4 5 6 7 8 9 11 13 15 17 19 21 23 25 27 29 31 33 35 37 39 41 43 45 47 49 51
Sequence Length (bp)

N22-LS

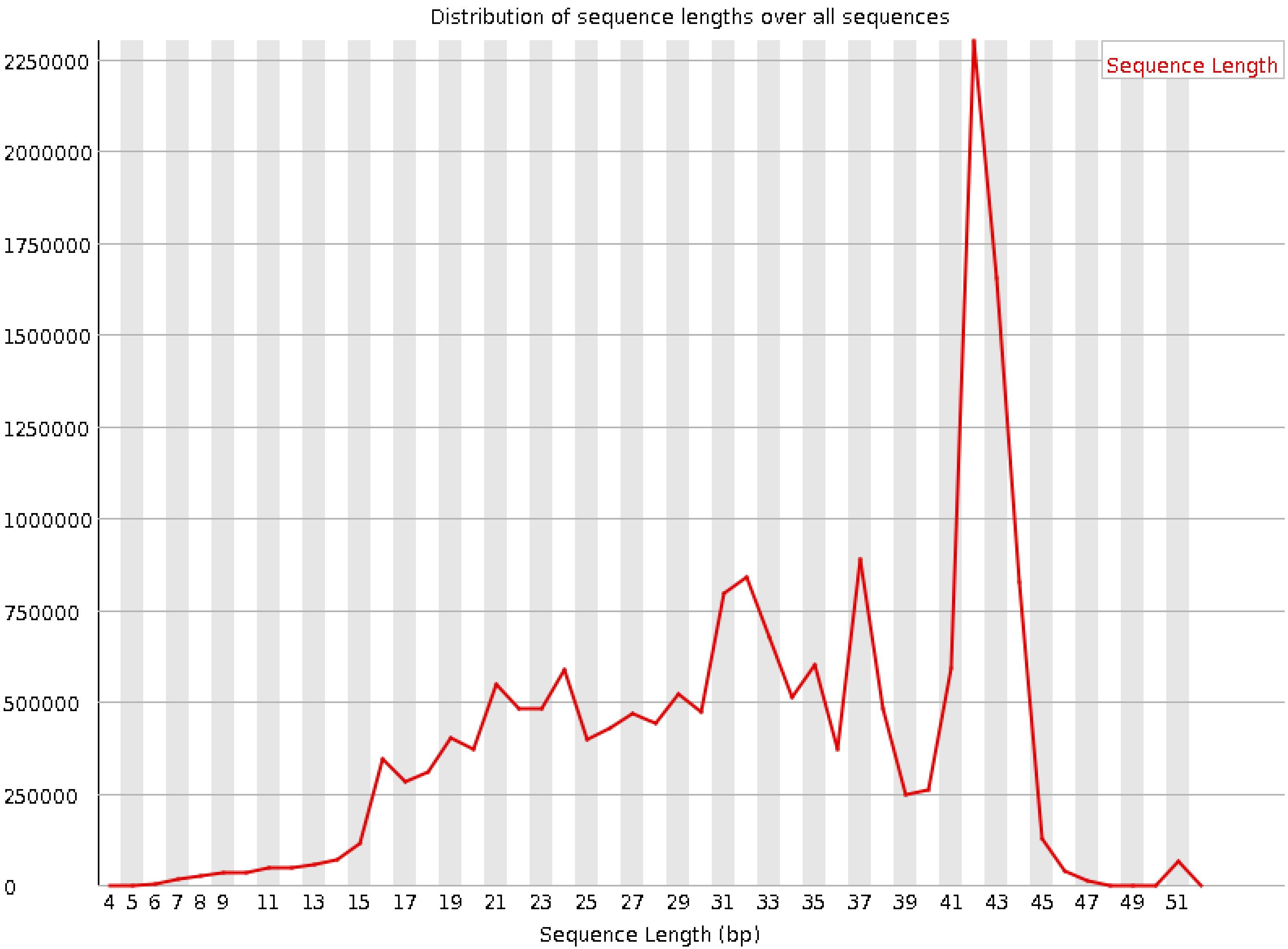
Distribution of sequence lengths over all sequences
Sequence Length
2250000
2000000
1750000
1500000
1250000
1000000
750000
500000
250000
0
4 5 6 7 8 9 11 13 15 17 19 21 23 25 27 29 31 33 35 37 39 41 43 45 47 49 51
Sequence Length (bp)

N22-LR

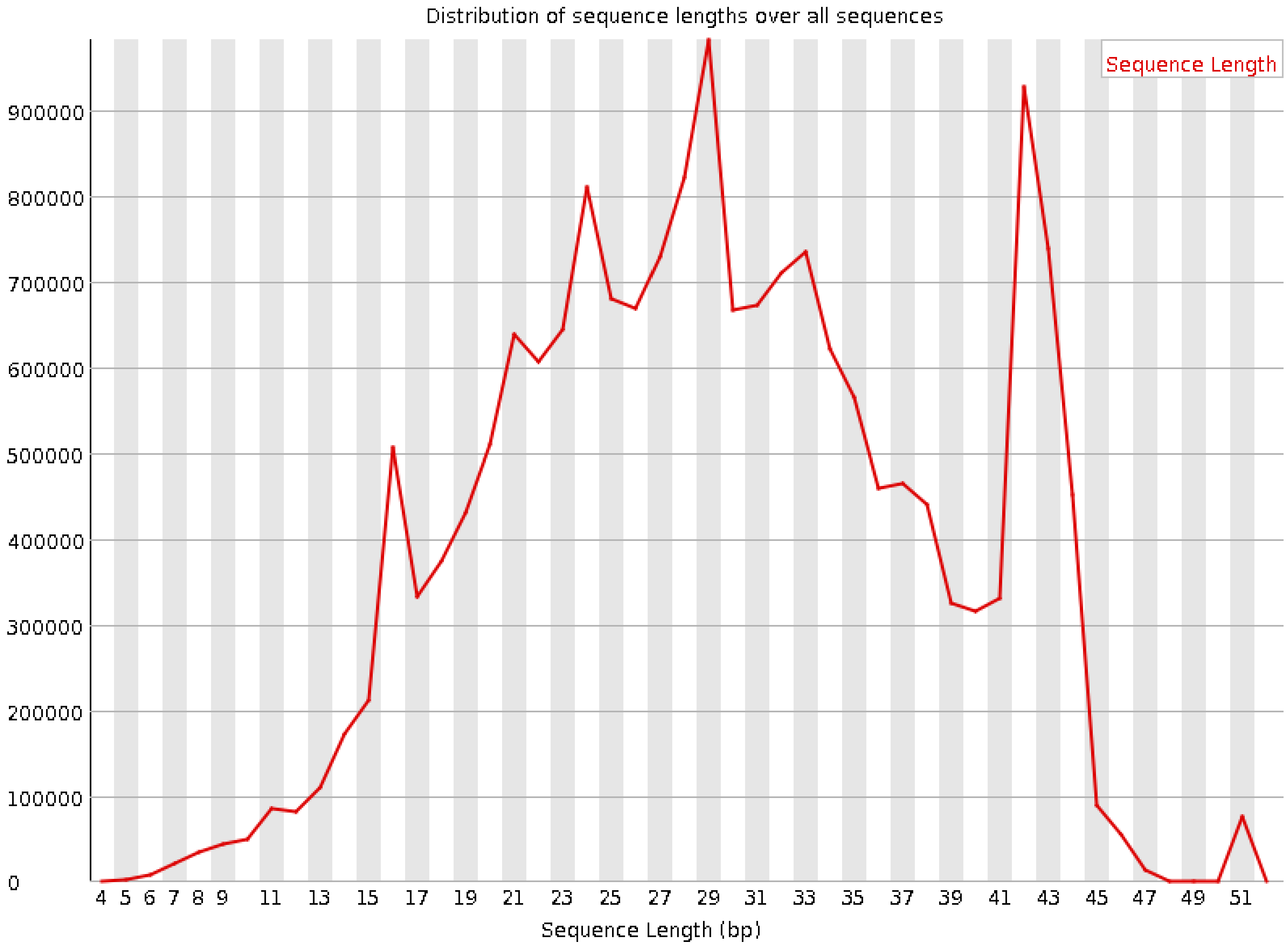
Distribution of sequence lengths over all sequences
Sequence Length
900000
800000
700000
600000
500000
400000
300000
200000
100000
0
4 5 6 7 8 9 11 13 15 17 19 21 23 25 27 29 31 33 35 37 39 41 43 45 47 49 51
Sequence Length (bp)

N22-SS

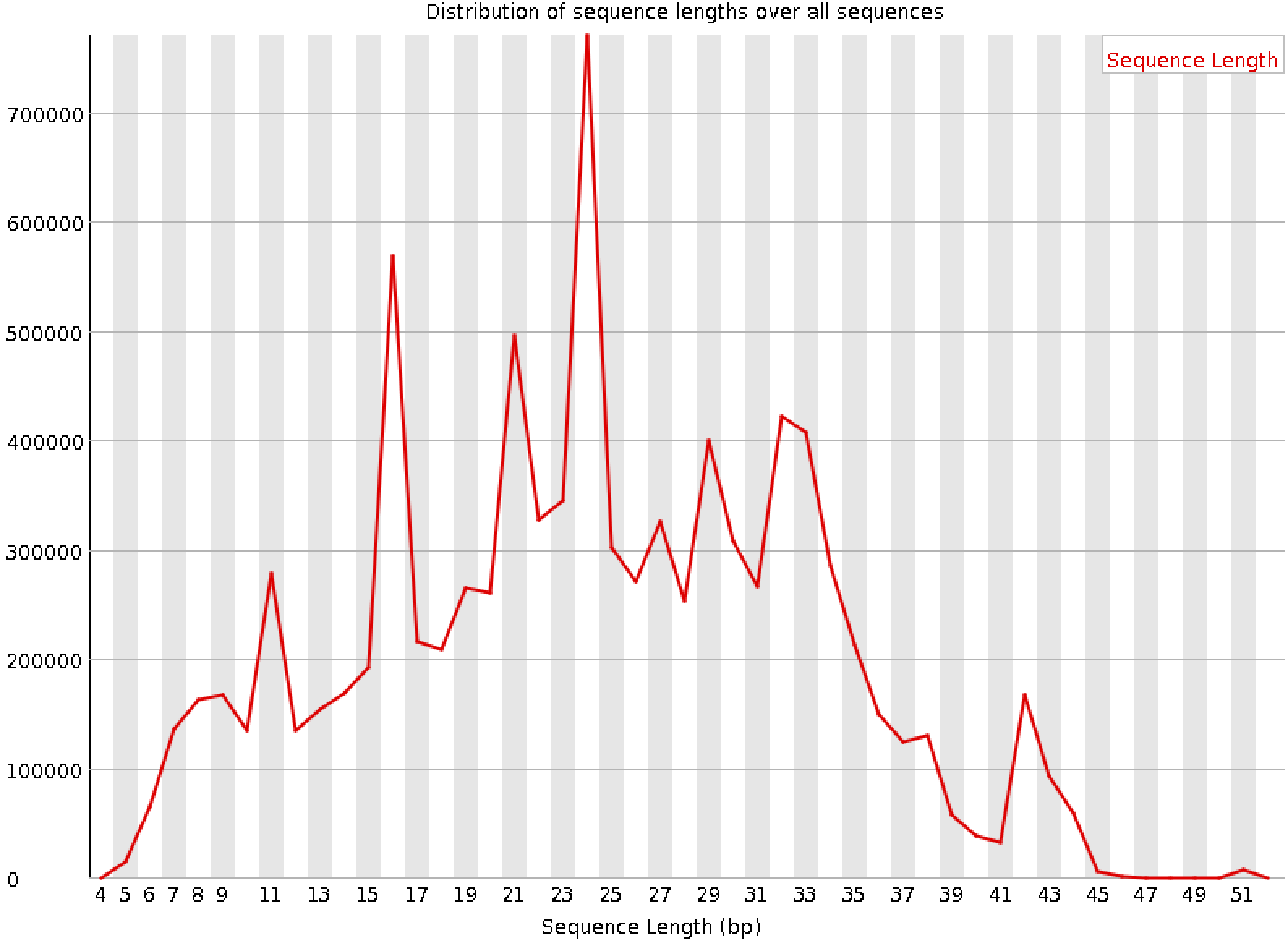

Distribution of sequence lengths over all sequences
Sequence Length
700000
600000
500000
400000
300000
200000
100000
0
4 5 6 7 8 9 11 13 15 17 19 21 23 25 27 29 31 33 35 37 39 41 43 45 47 49 51
Sequence Length (bp)

N22-SR

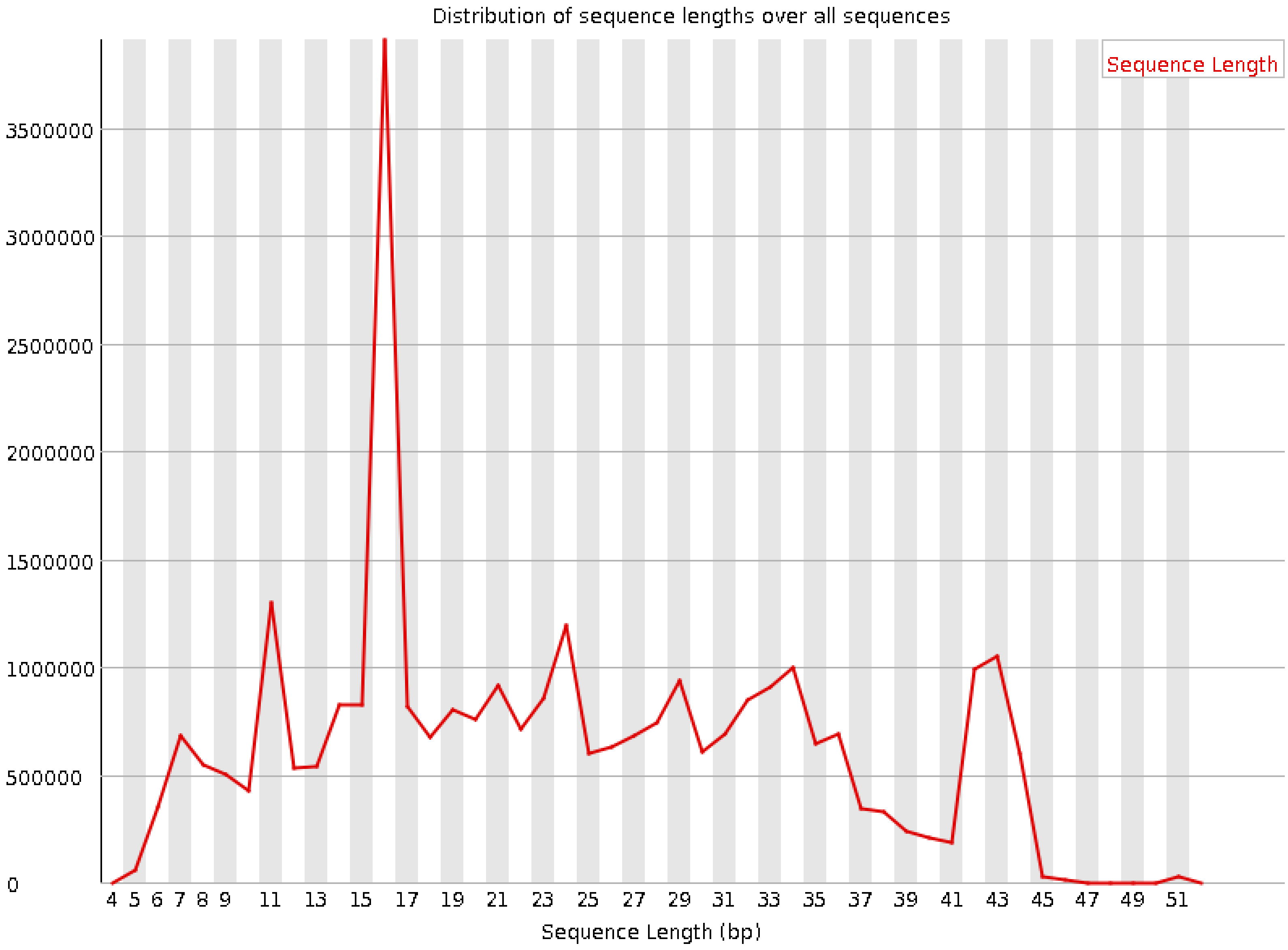

Distribution of sequence lengths over all sequences
Sequence Length
3500000
3000000
2500000
2000000
1500000
1000000
500000
0
4 5 6 7 8 9 11 13 15 17 19 21 23 25 27 29 31 33 35 37 39 41 43 45 47 49 51
Sequence Length (bp)

N22-RS

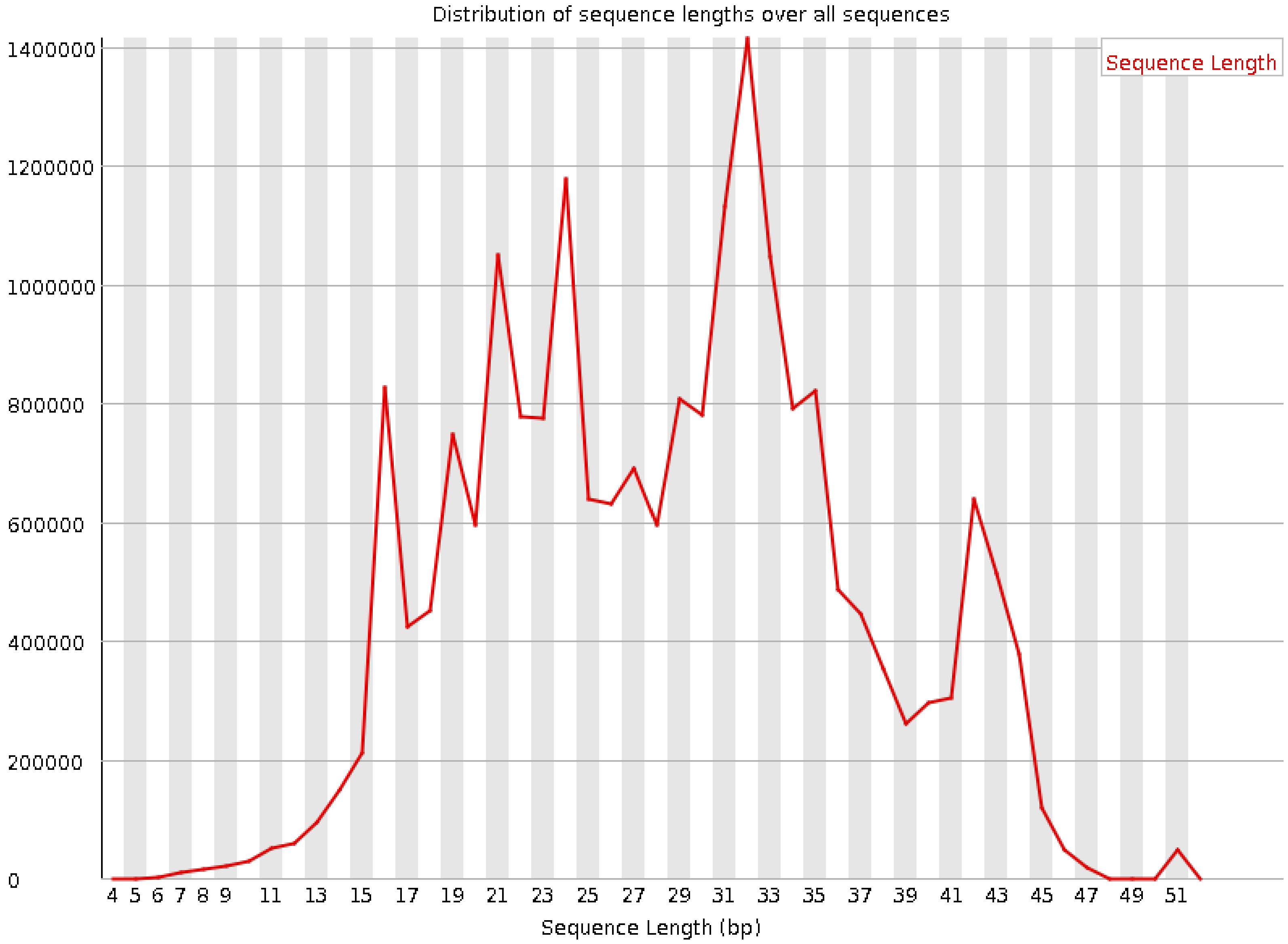

Distribution of sequence lengths over all sequences
Sequence Length
1400000
1200000
1000000
800000
600000
400000
200000
0
4 5 6 7 8 9 11 13 15 17 19 21 23 25 27 29 31 33 35 37 39 41 43 45 47 49 51
Sequence Length (bp)

N22-RR

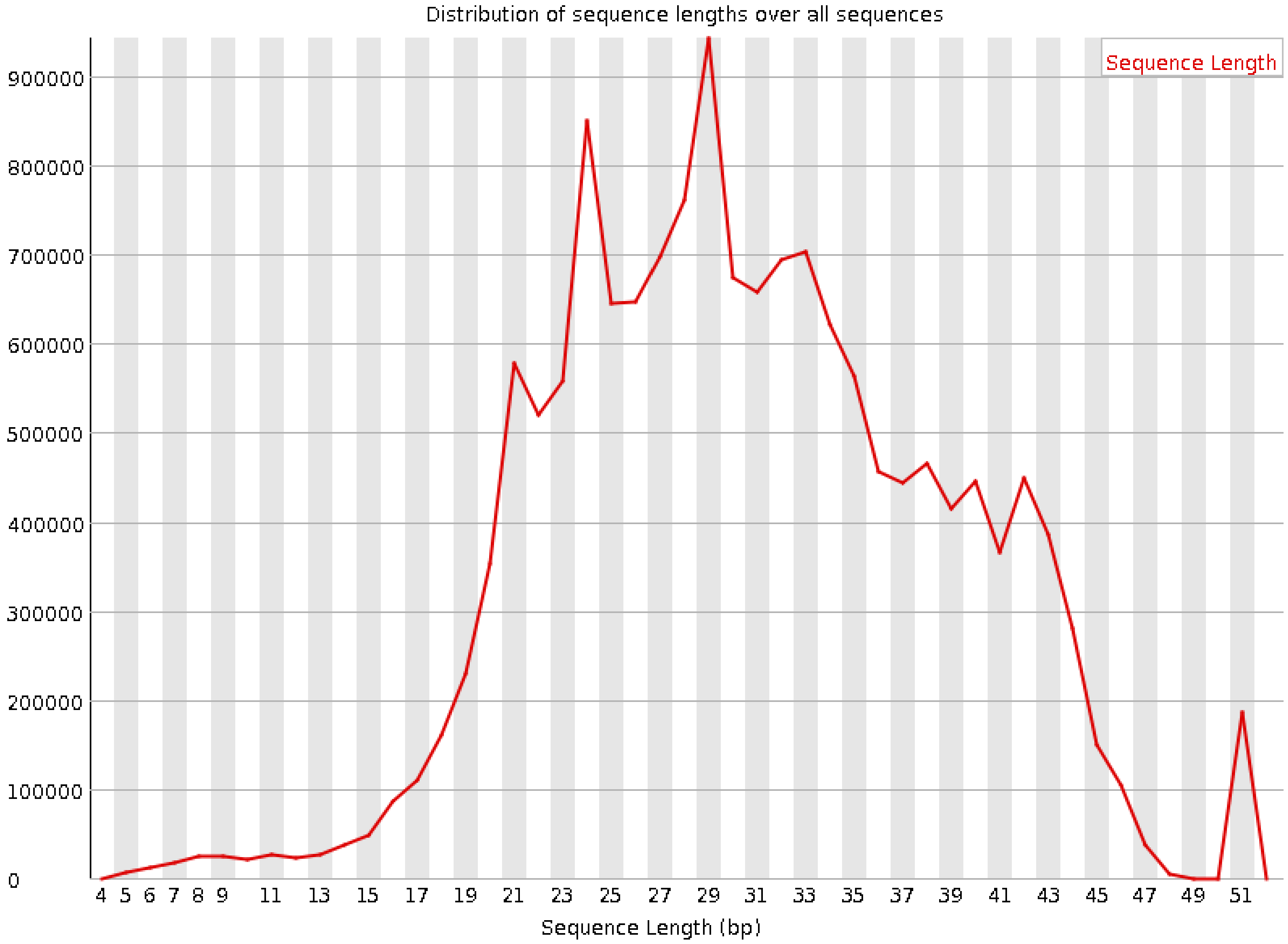
Distribution of sequence lengths over all sequences
Sequence Length
900000
800000
700000
600000
500000
400000
300000
200000
100000
0
4 5 6 7 8 9 11 13 15 17 19 21 23 25 27 29 31 33 35 37 39 41 43 45 47 49 51
Sequence Length (bp)
